# Serotonin promotes exploitation in complex environments by accelerating decision-making

**DOI:** 10.1186/s12915-016-0232-y

**Published:** 2016-02-04

**Authors:** Shachar Iwanir, Adam S. Brown, Stanislav Nagy, Dana Najjar, Alexander Kazakov, Kyung Suk Lee, Alon Zaslaver, Erel Levine, David Biron

**Affiliations:** The Institute for Biophysical Dynamics, The University of Chicago, Chicago, IL 60637 USA; Committee on Computational Neuroscience, The University of Chicago, Chicago, IL 60637 USA; Department of Physics and the James Franck Institute, The University of Chicago, Chicago, IL 60637 USA; Department of Genetics, The Alexander Silberman Institute of Life Sciences, The Hebrew University of Jerusalem, Jerusalem, Israel; Department of Physics and Center for Systems Biology, Harvard University, Cambridge, MA 02138 USA

**Keywords:** Patchy environments, Resource distribution, Foraging systems, Decision-making, Serotonin (5-HT), *C. elegans*, Serotonin, 5-HT, Foraging, Slowdown, Re-feeding

## Abstract

**Background:**

Fast responses can provide a competitive advantage when resources are inhomogeneously distributed. The nematode *Caenorhabditis elegans* was shown to modulate locomotion on a lawn of bacterial food in serotonin (5-HT)-dependent manners. However, potential roles for serotonergic signaling in responding to food discovery are poorly understood.

**Results:**

We found that 5-HT signaling in *C. elegans* facilitates efficient exploitation in complex environments by mediating a rapid response upon encountering food. Genetic or cellular manipulations leading to deficient serotonergic signaling resulted in gradual responses and defective exploitation of a patchy foraging landscape. Physiological imaging revealed that the NSM serotonergic neurons responded acutely upon encounter with newly discovered food and were key to rapid responses. In contrast, the onset of responses of ADF serotonergic neurons preceded the physical encounter with the food. The serotonin-gated chloride channel MOD-1 and the ortholog of mammalian 5-HT1 metabotropic serotonin receptors SER-4 acted in synergy to accelerate decision-making. The relevance of responding rapidly was demonstrated in patchy environments, where the absence of 5-HT signaling was detrimental to exploitation.

**Conclusions:**

Our results implicate 5-HT in a novel form of decision-making, demonstrate its fitness consequences, suggest that NSM and ADF act in concert to modulate locomotion in complex environments, and identify the synergistic action of a channel and a metabotropic receptor in accelerating *C. elegans* decision-making.

**Electronic supplementary material:**

The online version of this article (doi:10.1186/s12915-016-0232-y) contains supplementary material, which is available to authorized users.

## Background

The spatial distribution of resources in natural environments can be non-uniform. Patches of food sources generically result from non-linear interactions between multiple species and inhomogeneous environmental conditions [1–3]. As a result, foraging behavior in patchy environments is extensively studied both theoretically and experimentally in diverse species and habitats [4–10]. Yet, key questions such as the fitness consequences of efficient foraging and the neural basis of these behaviors are not sufficiently understood [11–15].

Food availability can affect locomotion patterns [16–19]. In the absence of food, *Caenorhabditis elegans* predominantly roam, a behavioral state characterized by fast, directional locomotion. In contrast, on a standard bacterial lawn *C. elegans* exhibit mostly non-directional dwelling and mean velocities are an order of magnitude lower than off food [18–21]. In addition, several minutes after being placed on food, starved *C. elegans* exhibit slower motion than well-fed animals. This was termed “enhanced slowdown”. The biogenic amine serotonin (5-hydroxytryptamine; 5-HT) modulates behaviors of *C. elegans* including locomotion, feeding, and egg laying [22–27] as well as the enhanced slowdown [16, 19, 28].

The dynamics of *C. elegans* locomotion during an encounter with newly discovered food have not been previously characterized. Under standard laboratory conditions, *C. elegans* typically forages on a large and homogeneously food-covered landscape. In such an environment responses to newly discovered food are not easily assayed and potential deficiencies may not incur a significant impact. In contrast, on a patchy foraging landscape responding quickly to a newly discovered patch of food may be crucial to efficient exploitation. Delayed reactions could potentially prove as detrimental as a deficiency in navigating to a patch in the first place.

Three neuronal cell types display robust serotonin biosynthesis in the hermaphrodite: the amphid sensory neuron ADF; the pharyngeal neurosecretory-motor neuron NSM; and the hermaphrodite-specific neuron HSN [29]. NSM was implicated in mediating an enhanced slowdown of locomotion on food after a period of starvation [16, 28, 30] and in decision-making during steady-state transitions between roaming and dwelling on food [20, 31]. The sensory neuron ADF has been primarily associated with navigation [32–36] and pharyngeal pumping [33, 37]. However, the specific roles of serotonergic neurons in mediating responses to newly encountered food are not well understood.

Here we show that serotonergic signaling accelerated the slowdown of animals upon encountering food, such that they could abruptly pause at the edge of a bacterial lawn. To address the biological relevance of an abrupt slowdown, we assayed exploration and resource exploitation of animals in patchy environments. Under these conditions, serotonergic signaling afforded a substantial advantage in exploitation. The pharyngeal neurosecretory-motor neuron NSM responded physiologically to the actual encounter and was the primary driver of the abrupt slowdown. In contrast, the onset of activity in the chemosensory serotonergic neuron ADF occurred prior to the encounter with food. Correspondingly, ADF affected locomotion during this time. Finally, we found that a 5-HT-gated chloride channel (MOD-1) and a 5-HT metabotropic receptor (SER-4) act together to accelerate *C. elegans* decision-making.

## Results

### The slowdown of *C. elegans* upon encountering food is abrupt

**Fig. 1:**
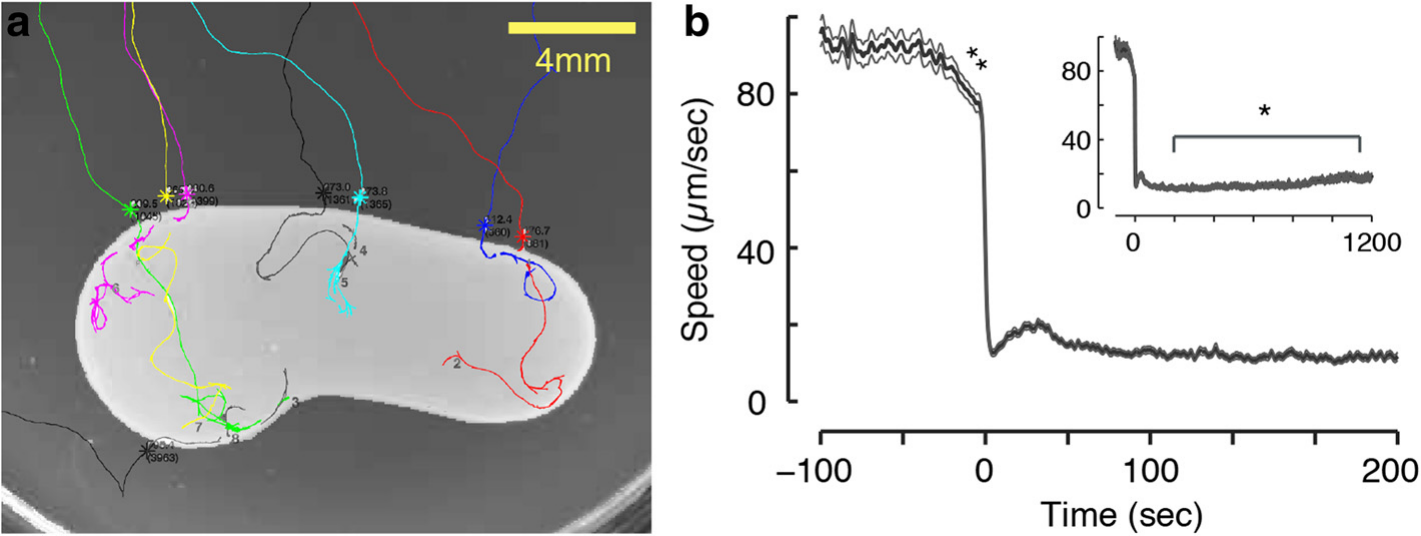
The slowdown of *C. elegans* upon encountering novel food is abrupt. **a** Typical trajectories of multiple tracked animals approaching a large bacterial lawn (light grey area). **b** The center of mass speed of tracked animals aligned to the time of encountering the edge of the bacterial lawn, t = 0 (mean ± standard error of mean; SEM, N = 288 animals). The mean deceleration during the 30-sec period that immediately preceded the encounter was 0.66 ± 0.04 μm/sec^2^ (double asterisks denote that it was significantly different from zero as determined by a t-test, *P* <0.01) with the edge of the bacterial lawn. Inset: the center of mass speed on food during intermediate and long times post-encounter. The mean speed at t = 1,100–1,200 sec was significantly higher than at t = 100–200 sec in agreement with previous reports (as determined by a t-test, asterisk denotes *P* <0.05)

Behavioral and physiological responses of *C. elegans* during encounters with newly found food were not previously characterized [16]. Under standard laboratory conditions, successful foraging does not depend on acute responses. However, on more complex terrain responding to discovery in a timely fashion may prove as important as the ability to navigate towards the food source. To address this, we used continuous video recordings to resolve the dynamics of locomotion upon re-feeding after a period off food with a high temporal resolution (Fig. 1a).

The most striking feature of the observed dynamics was an abrupt slowdown upon encountering the edge of the bacterial lawn: a nearly complete halt reached within 3 sec (τ = 1.73 ± 0.12 sec, Fig. 1b), where the timescale τ was obtained from a fit to a single exponential decay. After a brief pause, wild-type animals typically advanced about one body length into the large bacterial lawn during a 1-min period. During the following 10–15 min, animals predominantly dwelled before reaching steady-state transition rates between roaming (fast, mostly forward locomotion) and dwelling [20, 38]. The transiently augmented dwelling behavior was consistent with previous reports of enhanced slowing [16, 18].

Immediately preceding the encounter with the edge of the bacterial lawn, the roaming animals exhibited a mild but significant deceleration. During the 30-sec period preceding the encounter, the mean deceleration was 0.66 ± 0.04 μm/sec^2^, *P* <0.01 (Fig. 1b and Additional file 1: Movie S1). Despite being small in magnitude, this mild preemptive slowdown was observed in >70 % of the individual wild-type encounters (Additional file 2: Figure S1D). The overall structure of the response to encountering food was conserved across a range of experimental conditions. In particular, characteristics of motion during the encounter and the following 15 min were only weakly affected by or independent of the time the animals spent off food prior to the assay (Additional file 2: Figure S1). Thus, a robust and eminent feature of the response to newly discovered food was a rapid slowdown upon encounter with the edge of the lawn.

### Serotonergic signaling accelerates slowdown upon re-feeding

**Fig. 2:**
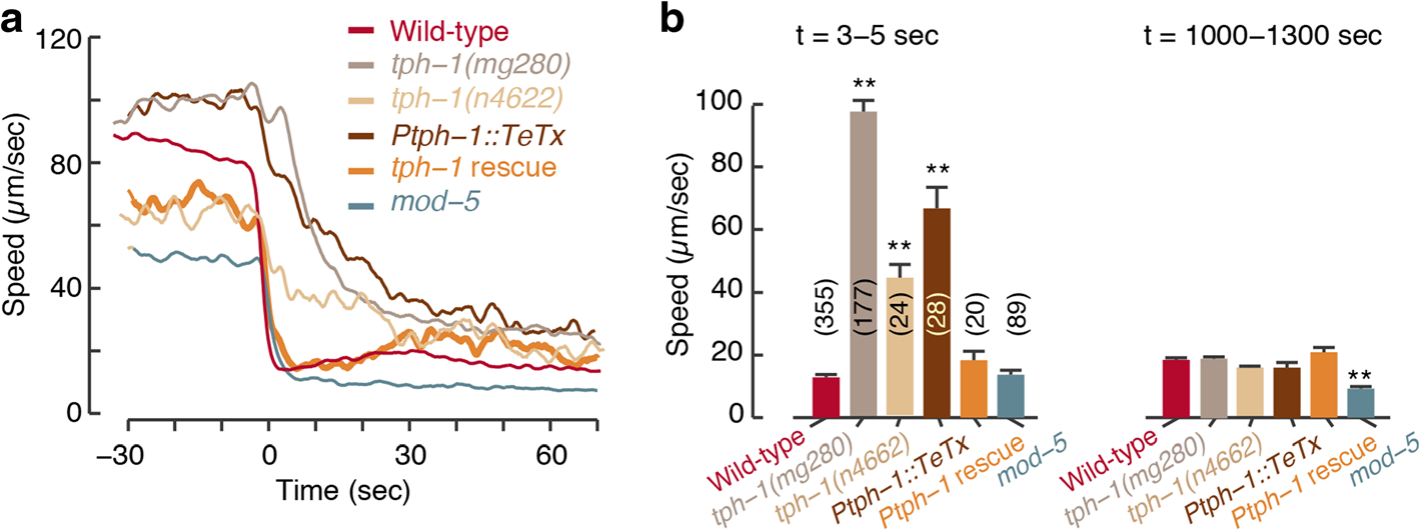
Serotonergic signaling accelerates slowdown upon re-feeding. **a** The mean speeds of wild-type 5-HT-deficient strains around the time of encounter with the edge of a large bacterial lawn (shown without standard errors as a guide to the eye; see summary statistics in next panel). **b** The speeds of the strains shown in panel (**a**) shortly after the encounter and 20 min later. Twenty minutes after the encounter, wild-type and *tph-1* center of mass speeds were similar. Bars depict mean ± SEM and the number of animals assayed for each strain is noted in parentheses. Mean velocities were compared to wild-type using an ANOVA test and corrected post hoc for multiple comparisons using Tukey’s honest significant difference (HSD) test. Double asterisks denote a significant difference from wild-type (*P* <0.01). For *Ptph-1::TeTx* and *Ptph-1::tph-1*, three independent transgenic lines were assayed and one representative dataset is shown

We next asked whether serotonin might affect the timescale of slowdown upon encountering an edge of a bacterial lawn [16–19]. To test this, we assayed mutants in which the synthesis of 5-HT was abolished due to the loss of function of the tryptophan hydroxylase TPH-1 [39]. Upon re-feeding, *tph-1* mutants exhibited a significant increase of the timescale of slowdown: τ = 14.49 ± 0.39 sec and τ = 30.80 ± 1.72 sec for *tph-1(mg280)* and *tph-1(n4622)*, respectively. In our hands, this was the most prominent phenotype resulting from loss of 5-HT synthesis and center of mass speeds of *tph-1* mutants were not higher than wild-type 20 min after the encounter (see Fig. 2 and Additional file 3: Figure S2). Expressing the functional *tph-1* gene under its innate promoter successfully rescued the defect, i.e. restored wild-type-like rapid responses (τ = 2.89 ± 0.17 sec, Fig. 2a, b).

Animals in which vesicle exocytosis was blocked by the tetanus toxin light chain gene [40, 41] in all of the serotonergic neurons (*Ptph-1::TeTx*) were phenotypically similar to *tph-1* mutants (τ = 24.90 ± 0.56 sec, Fig. 2 and Additional file 3: Figure S2). Mutants in the *mod-5* gene, encoding a transporter required for 5-HT re-uptake, exhibited a lower mean speed both off and on food (see also [16, 42]) and an abrupt slowdown upon encounter (τ = 3.24 ± 0.14 sec, Fig. 2 and Additional file 3: Figure S2). To test the impact of differences in baseline locomotion, we confirmed that the changes in relative velocity maintain the phenotypic trends observed in the wild-type, mutant, functional ablation, and rescue strains assayed (Additional file 4: Figure S3). Taken together, these results indicate that 5-HT signaling accelerates slowdown upon encountering food and acts on a timescale of 2–3 sec.

### An abrupt slowdown supports exploitation of a small patch of food

Under what circumstances could an abrupt slowdown be advantageous? A gradual modulation of locomotion can suffice when the spatial scale of the feeding area is much greater than the length scale associated with the slowdown. In contrast, in environments where food is spatially confined to small patches, an abrupt slowdown may increase exploitation [3, 14, 43]. The ratio between the typical speed of a roaming animal and the timescale of slowdown determined the relevant length scale, which was comparable to one body length.

To test the utility of abrupt slowdown, we placed animals on plates with patches of bacterial food that were 0.5–1 mm in diameter, termed “micro-patches”. This assay was repeated using different concentrations of food and various patch arrangements (see Methods). We measured the fraction of time spent on a patch of food during the 5 min following the first encounter with this patch. Responses were scored visually and divided into four categories, where the first two resulted in favorable exploitation of the food patch (see Methods).

We assayed *tph-1* mutants and *Ptph-1::TeTx* transgenics in the presence of micro-patches. In both cases the animals spent significantly less time on a patch of food during the 5 min following the first encounter with this patch, as compared to wild-type (Additional file 5: Figure S4A and Additional file 6: Movie S2). The chances of exploitation increased with the concentration of food on the patch in a dose-dependent manner in wild-type animals, *tph-1* mutants, and *Ptph-1::TeTx* transgenics. Serotonin-deficient animals were completely unable to stop at patches of low food concentrations and performed poorly as compared to wild-type under all of the conditions tested. Thus, a deficiency in either the synthesis or the release of 5-HT reduced the efficiency of translating an encounter with a small patch of food to successful exploitation in a concentration-dependent manner.

### Serotonergic signaling promotes exploitation in a complex environment

**Fig. 3:**
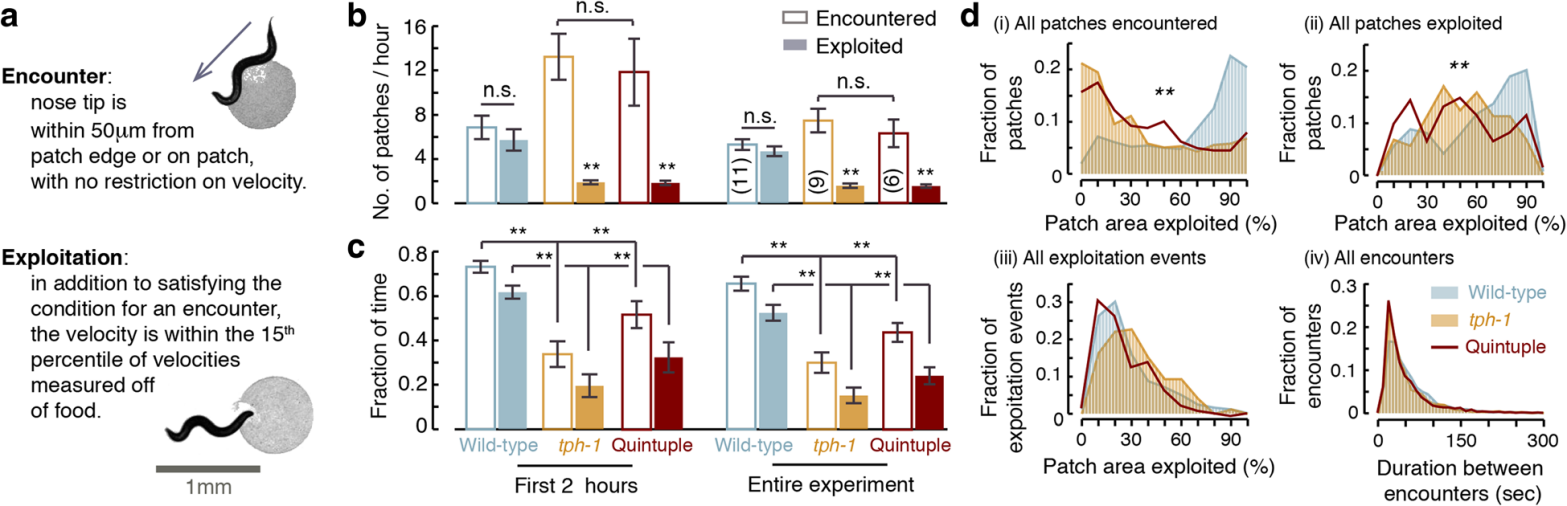
Serotonergic signaling promotes exploitation in a complex environment. Wild-type animals and *tph-1* mutants were assayed on a square lattice arrangement of 49 micro-patches (see also Additional file 5: Figure S4B). **a** Frames in which the nose of the animal was on a patch were labeled “encountered”. Frames were labeled “exploited” once, in addition, sub-threshold speed was measured (see Methods). Top, a *tph-1* mutant encountering a patch; bottom, a wild-type animal exploiting a patch. **b** The mean number of micro-patches encountered and exploited during the first 2 hours or the entire duration of each assay. **c** The mean fraction of time spent on encounters or exploitation during the first 2 hours or the entire duration of each assay. When animals were not encountering or exploiting they were moving (predominantly roaming) off food between the patches. In panels (**b**, **c**), the number of animals assayed for each strain is noted in parentheses, error bars depict SEM, means were compared using an ANOVA test corrected post hoc for multiple comparisons using Tukey’s HSD test, and double asterisks denote a significant difference (*P* <0.01). By both measures, *tph-1* mutants were deficient as compared with wild-type. **d** The percentage of the area exploited during the assay was calculated for each micro-patch that was encountered at least once. (i) A histogram of exploited percentages for all encountered patches. (ii) A histogram of exploited percentages for all exploited patches (excluding patches that were only encountered). (iii) A histogram of exploited percentages for single continuous periods of exploitation (termed “events”). (iv) A histogram of the durations between consecutive encounters. The mutants encountered as many patches as wild-type and did not exploit a smaller area of the patch during a single event. However, once they veer off the patch or fully consume it, *tph-1* mutants are deficient in initiating the next exploitation event and thus exhibit lower cumulative exploitation. The distributions were compared to wild-type using the *k*-sample Anderson–Darling test and double asterisks denote a significant difference (*P* <0.01)

Exploitation on a patchy landscape can be affected not only by first encounters, but also by various (possibly compensatory) factors including feeding behavior once on food or differences in foraging patterns. Thus, a first encounter phenotype may not translate in a straightforward manner to a competitive advantage. To test this, we placed the animals in an arena where 49 micro-patches were positioned in a square lattice arrangement and monitored their behavior for 5–10 hours (Additional file 7: Movie S3 and Additional file 8: Movie S4). Although fast locomotion does not entirely preclude feeding, small deposits of food are more efficiently exploited when the animals move very slowly on the patch. Therefore, in these assays, detectable exploitation events were defined when two conditions were met concurrently: the mouth of the animal was on a patch of food; and its velocity during the encounter was below an experimentally determined threshold (see Fig. 3a and Methods).

To examine the role of 5-HT signaling in exploiting a complex environment, we assayed wild-type animals, *tph-1* mutants, and quintuple mutants lacking the function of the five serotonin-binding receptors that have been identified in *C. elegans* [25, 26]. Two simple measures of the efficiency of food exploitation are: 1) comparing the numbers of patches that were exploited with those merely encountered; and 2) comparing the overall fraction of time spent at patch locations to the time spent exploiting. Both measures were quantified during the first 2 hours of the experiment, when the environment was mostly intact, and throughout the experiment, as patches were progressively consumed (Fig. 3b, c and Additional file 5: Figure S4B–D).

While wild-type animals exploited as many patches as they encountered, *tph-1* mutants were unable to exploit the vast majority of encountered patches. Moreover, these mutants spent significantly less time than wild-type at patch locations, whether they were regarded as exploiting or not. Thus, even if animals fed efficiently without slowing, for example by frequent turns resulting in multiple passes through a patch, serotonergic signaling remained advantageous.

Next, we measured the fractions of the area of a food patch that was encountered or exploited (Fig. 3d and Additional file 5: Figure S4C). When all encountered patches (throughout the experiment) were considered, wild-type animals covered a significantly larger fraction per patch than *tph-1* mutants (Fig. 3d, i). When we limited the analysis to exploited patches, the difference between wild-type and *tph-1* mutants was smaller but remained significant (Fig. 3d, ii), indicating that *tph-1* mutants were capable of exploiting patches once exploitation was initiated.

Possible contributors to deficient exploitation could have been a smaller coverage of the patch per event due to enhanced patch leaving [44], an exploration defect resulting in fewer encounters, or the defect in initiating exploitation upon encounter demonstrated above. As depicted in Fig. 3d, iii, *tph-1* mutants exploited a similar area per event, suggesting that elevated patch leaving did not appreciably contribute to reduced exploitation. The time spent between encountering patches was also similar between *tph-1* and wild-type animals (Fig. 3d, iv), indicating that the mutants did not exhibit exploration/navigation defects. Combined, our various assays suggested that a major contributor to the *tph-1* exploitation defect was their inability to slow down abruptly, and that this defect was not sensitive to experimental details. Moreover, mutants lacking the function of all five *C. elegans* serotonin receptors were significantly defective as compared to wild-type and similar to *tph-1* mutants (Fig. 3b–d). Thus, serotonergic signaling conferred an exploitation advantage in a complex environment by accelerating decision-making.

### The serotonergic neurons ADF and NSM respond prior to and upon encountering food, respectively

Spontaneous physiological activity of NSM on food is sporadic [20] and chemical cues can evoke slow changes in NSM cytosolic calcium [45]. In ADF, familiar food and salt can evoke calcium transients [33, 46]. However, the activity of serotonergic neurons was not examined during encounters with newly discovered food. To characterize the physiological activity that promotes the rapid responses we observed, we monitored calcium levels in freely behaving animals using the genetically encoded indicator GCaMP3.0 [47]. Our behavioral assays indicated that *egl-1* mutants, lacking the HSN serotonergic neuron type [48, 49], were indistinguishable from wild-type (data not shown). Therefore, to study cellular mechanisms, we focused on the physiological activity in NSM and ADF prior to, during, and after re-feeding encounters.

**Fig. 4:**
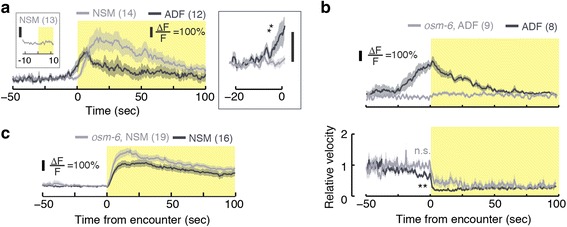
The serotonergic neurons ADF and NSM respond physiologically during re-feeding. **a** Left: traces of fluorescence from NSM::GCaMP (grey) and ADF::GCaMP (black) transgenics re-feeding on a large bacterial lawn. The time of encountering the edge of the lawn was defined as t = 0 . Plots depict mean ± SEM and the number of animals assayed for each strain is noted in parentheses. Inset: NSM was not activated when the animals abruptly paused due to gently colliding with an obstacle. The yellow shading denotes the time following the encounter (t >0 ). Right: a zoomed view of the calcium transients shortly prior to the encounter. Fluorescence in ADF but not NSM neurons was significantly higher than baseline during the 5 sec preceding the encounter (mean intensities were compared to their respective baselines using a t-test, *P* <0.01). (**b**, **c**) GCaMP fluorescence in ADF and NSM in *osm-6* mutant backgrounds and their respective wild-type background control groups using an improved imaging system (see Methods). The velocity of the tracked ADF neurons was reduced prior to the encounter in the control group but not in *osm-6* mutants (panel (**b**), bottom, t-test, *P* <0.01). NSM activity was not abolished by the mutation

NSM neurons of food-deprived animals were strongly activated at the time of the encounter with the edge of a bacterial lawn (t = 0 , Fig. 4). Calcium levels peaked 15–20 sec after the encounter and remained above baseline for >100 sec. In contrast, ADF neurons responded up to 25 sec prior to the arrival of the animals at the edge of the lawn (see Fig. 4, Additional file 9: Movie S5, and Additional file 10: Movie S6) and returned to baseline calcium levels after 50 sec. The early activation of ADF was likely due to its known role as a chemosensory neuron [32–35].

To further address the activation of ADF, we assayed its physiological activity in *osm-6* mutants. The *osm-6* gene encodes an intraflagellar transport component. Its absence results in defects to the ultrastructure of sensory cilia and, as a result, in chemosensory and mechanosensory defects. Expression of *osm-6* was reported in many ciliated neurons, including ADF, but not in NSM [50, 51]. In these assays, we observed a prominent activation of ADF prior to the encounter in control animals and no activation of ADF at any time in *osm-6* mutants. Correspondingly, the preemptive slowdown was significant in the control group and absent in the mutants (Fig. 4b). In contrast, NSM responses were not prominently affected by the loss of OSM-6 function (Fig 4c). Thus, chemosensation by ADF contributes to the preemptive slowdown.

Physiological responses of ADF to bacteria supernatant were detected in a microfluidic device [52]. To demonstrate ADF responses to diffusible chemicals from food in freely behaving animals on a standard assay plate, we recorded from animals placed on the face of an agar pad opposing the side supporting the bacterial lawn (see Methods). The minimal possible distance between animals thus placed and the bacterial lawn was 2–3 mm, as determined by the thickness of the pad. Although this assay does not reproduce the geometry of a gradient near the edge of a lawn, it effectively serves to isolate the diffusible chemicals from the bacteria that produce them. A mock encounter was defined as the time when the position of the animal was directly above the edge of the lawn, and activity during the 20 sec surrounding the mock encounter was compared to baseline (Additional file 11: Figure S5A). Consistent with chemosensation, we observed activation of ADF, but not of NSM, in the vicinity of the mock encounter in the reverse patch assay.

The abrupt slowdown was completed in 2–3 sec, while the calcium transients in NSM and ADF lasted for tens of seconds. However, *tph-1* mutants and *Ptph-1::TeTx* transgenics slowed down over >30 sec (Additional file 11: Figure S5B). Moreover, NSM calcium transients predicted dwelling states for several minutes on bacterial lawns [20] and the average time spent by a wild-type animal on a small patch was similarly long (see Fig. 3b, c). Taken together, our measurements demonstrated that ADF and NSM responded physiologically to the proximity and encounter with bacterial food, respectively, and continued to affect locomotion for tens of seconds after the encounter.

Is NSM required for exploiting food resources or for generic motor control? To address this we monitored the activity of NSM when the animal paused for a reason other than food encounters, i.e. in order to avoid an obstacle. We positioned a standard platinum pick near the anterior of a forward moving animal, such that the animal gently touched it due to its own motion. These collisions typically evoked abrupt pauses followed by brief reversals. In this case, NSM neurons were not detectably activated (Fig. 4a, left inset). Thus, NSM is not part of a general motor circuit that controls slowing down. Rather, it underlies a specific subset of behaviors including responses that facilitate exploitation of localized food resources.

### ADF and NSM play complementary roles in mediating the dynamics of slowdown

We observed that ADF, but not NSM, was activated before the animal reached the edge of the bacterial lawn. The timing of ADF activation resembled the timing of the mild preemptive slowdown prior to the encounter (Fig. 1b and 4). Both the abrupt slowdown upon encounter and the mild preemptive slowdown were affected by changes in 5-HT signaling (Fig. 2 and Additional file 12: Figure S6), suggesting that 5-HT may regulate distinct aspects of the overall response. To explore the individual contributions of different serotonergic neurons, we assayed transgenics expressing TeTx in either NSM or ADF.

**Fig. 5:**
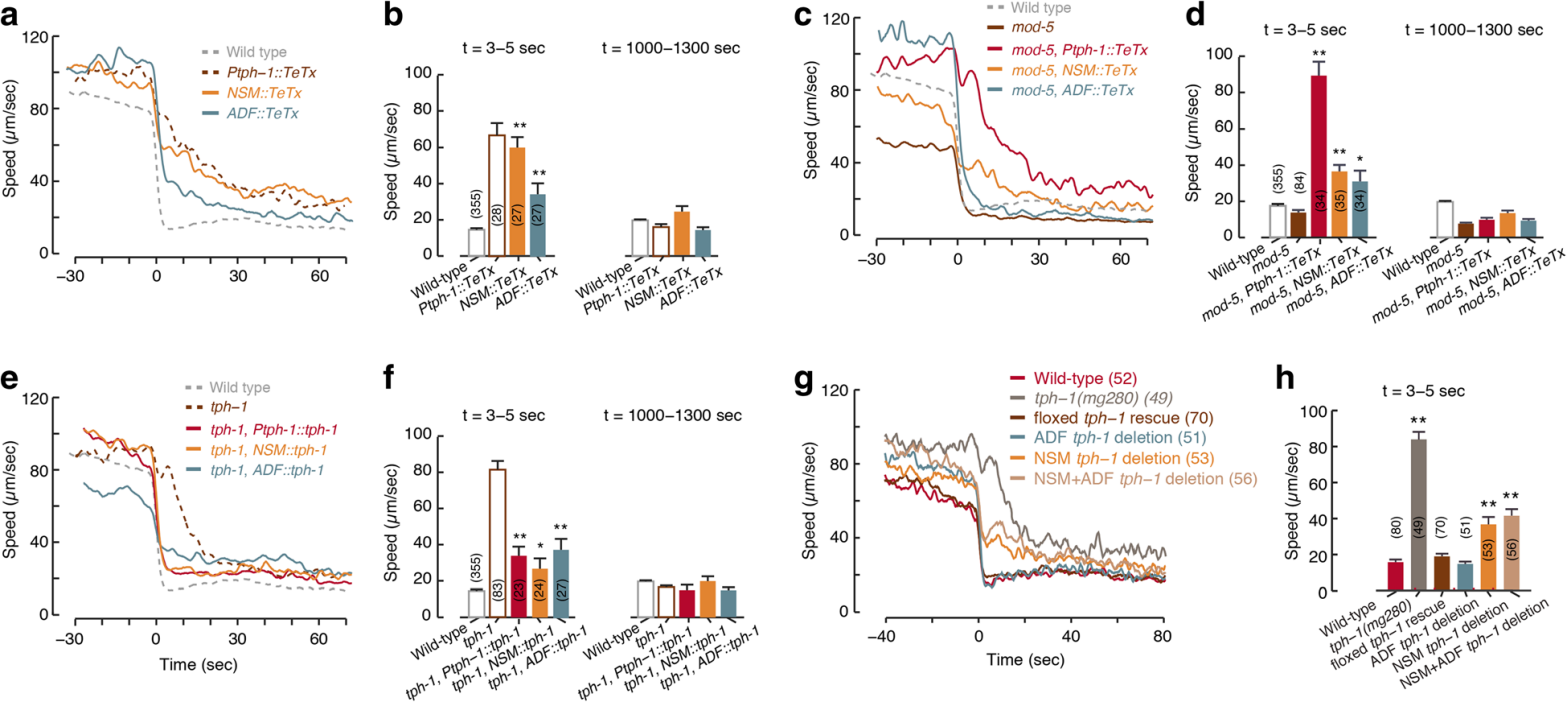
Both ADF and NSM serotonergic neuron types affect the dynamics of slowdown. **a** The mean speeds of transgenics in which NSM or ADF have been genetically silenced. The time of encounter with a large bacterial lawn was defined as t = 0 . Traces are shown without standard errors as a guide to the eye; see summary statistics in next panel. **b** The speeds of the strains shown in panel (**a**) shortly after the encounter and their baseline speeds on food 20 min later. Bars depict mean ± SEM and the number of animals assayed for each strain is noted in parentheses. Double asterisks denote a significant difference from wild-type, *P* <0.01. Data from wild-type and *Ptph-1::TeTx* strains was reproduced from Fig. 2 and shown as dashed lines and empty bars for comparison. (**c**, **d**) Same as panels (**a**, **b**), except that the genetic silencing was performed on a *mod-5* mutant background. (**e**, **f**) Same as panels (**a**, **b**) for transgenics in which the intact *tph-1* gene was rescued in NSM, in ADF, or in both. Combined, genetic silencing and rescue assays implicate both neuronal types in mediating the observed behavior. (**g**, **h**) Same as panels (**a**, **b**) for Cre-mediated deletions of the *tph-1* genes (see [20]). In all bar plots, mean velocities were compared to wild-type using an ANOVA test and corrected post hoc for multiple comparisons using Tukey’s HSD test. Single and double asterisks denote a significant difference from wild-type (*P* <0.05 and *P* <0.01, respectively). Three independent transgenic lines were assayed in the cases of *NSM::TeTx*, *ADF::TeTx*, *NSM::tph-1*, and *ADF::tph-1*. One of each of the *TeTx* lines (*Ptph-1::TeTx*, *NSM::TeTx*, and *ADF::TeTx*) was crossed to a *mod-5(n3314)* mutant background and similarly assayed. One line was assayed for each floxed *tph-1* deletion strain [20]

Animals expressing TeTx in NSM but not in ADF slowed down gradually upon encounter (τ = 25.65 ± 1.18 sec), similarly to *Ptph-1::TeTx* animals and *tph-1* mutants, but retained the mild preemptive slowdown. In contrast, the preemptive slowdown was abolished in animals expressing TeTx in ADF but not in NSM, while their abrupt slowdown upon encounter was more mildly defective (τ = 7.62 ± 0.39 sec, Fig. 5a, b and Additional file 4: Figure S3). These trends were maintained on a background of the loss of function of the 5-HT transporter MOD-5 (Fig. 5c, d and Additional file 4: Figure S3). In addition, functional ablation of either NSM or ADF resulted in partial phenotypes with respect to foraging in a complex environment (Additional file 5: Figure S4C). Thus, ADF and NSM may play complementary roles in mediating locomotion during foraging.

To assay the sufficiency of ADF and NSM, we expressed *tph-1* specifically in NSM or ADF on a *tph-1(mg280)* mutant background. Rescuing the function of TPH-1 in NSM mostly restored the abrupt slowdown upon encounter (τ = 2.49 ± 0.16 sec) but not the preemptive slowdown. ADF-specific rescues resulted in an opposite phenotype (Fig. 5e, f and Additional file 12: Figure S6). High expression levels typical of such experiments may have contributed to the observed phenotypes by allowing one neuronal type to partially compensate for the absence of the other. Cell-specific *tph-1* deletion transgenics [20] exhibited similar, albeit weaker, phenotypes (Fig. 5g, h, Additional file 4: Figure S3, Additional file 5: Figure S4D, and Additional file 12: Figure S6). Incomplete penetrance of Cre expression or inactivation of the floxed *tph-1* gene could not be ruled out as a possible contributor to these weaker phenotypes. Taken together, these results indicate that NSM and ADF act through 5-HT signaling to mediate slowdown prior to and during encounters.

### Optogenetic activation of serotonergic neurons induces a rapid slowdown off food

Optogenetic activation of serotonergic neurons was previously shown to affect locomotion on food [20] and off food [28]. To assess the sufficiency of 5-HT signaling in mediating abrupt slowdown, we optogenetically activated the serotonergic neurons of freely moving starved animals using the light-activated cation channel channelrhodopsin (ChR2). Innate responses of *C. elegans* to blue light were avoided by performing the assays on a light-insensitive (*lite-1*) mutant background [53, 54].

**Fig. 6:**
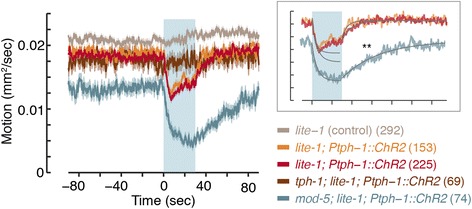
Optogenetic activation of serotonergic neurons induces a rapid slowdown off food. The temporal dynamics of motion were assayed using the frame difference method (i.e. the number of pixels that changed their value between consecutive frames of a movie of a single animal; see Methods). Three independent lines (two *lite-1* backgrounds and one *lite-1; mod-5* background) expressing ChR2 in both NSM and ADF serotonergic neurons (*Ptph-1::ChR2*) were assayed. Control animals (*lite-1*) lacked ChR2 or TPH-1. The shaded area depicts the period in which the blue light was on. Inset: single exponential fits to each dataset (see Methods). Plots depict mean ± SEM, the number of stimuli assayed is noted in parentheses for each condition, the recovery timescale of *lite-1; mod-5* double mutants was compared to that of *lite-1* mutants using an ANOVA test corrected post hoc for multiple comparisons using Tukey’s HSD test, and double asterisks denote significant differences (*P* <0.01)

Upon exposure to a pulse of blue light, the locomotor activity of *lite-1;* Ex[*Ptph-1::Chr2*] animals declined by 35 % as measured by the frame difference method [55], and remained low for the duration of the pulse (Fig. 6). The timescales of the decline were 3.6 ± 0.5 sec and 5.2 ± 0.9 sec in the two lines that were assayed. After the blue light was turned off, locomotor activity returned to baseline levels on timescales of τ = 5.4 ± 0.6 sec and τ = 10.1 ± 0.6 sec, respectively (Fig. 6, inset). Animals not expressing ChR2 assayed in the presence of the co-factor all-trans-retinal (ATR) did not detectibly respond to blue light. Importantly, activating the serotonergic neurons on a *tph-1* mutant background (*tph-1; lite-1;* Ex[*Ptph-1::ChR2*]) did not evoke a detectable slowdown response (Fig. 6). This indicated that 5-HT signaling was required for the observed responses.

To further investigate the role of 5-HT, we assayed *mod-5* mutants. Exposure to blue light reduced the locomotor activity of *mod-5; lite-1;* Ex[*Ptph-1::ChR2*] animals by 70 %. Locomotion of *mod-5* mutants declined throughout the 30-sec light stimulus (τ = 5.1 ± 1.1 sec) and returned to baseline post-stimulation slower than wild-type (τ = 37.3 ± 2.1 sec, *P* <0.01, see Fig. 6). This suggested that the uptake of endogenous 5-HT also contributed to shaping the modulation of locomotion on short timescales. Combined with our earlier results, these observations associate the dynamics of 5-HT signaling and of slowdown.

### Efficient exploitation on a patchy landscape is mediated through the synergistic action of the 5-HT-gated channel MOD-1 and the 5-HT metabotropic receptor SER-4

Five serotonin-binding receptors have been identified in *C. elegans* [25, 26]. Of these, the G_o_-protein coupled receptor SER-4 (an ortholog of mammalian 5-HT1 receptors) and the serotonin-gated chloride channel MOD-1 have been implicated in slowing locomotion in response to exogenous 5-HT [28]. However, endogenous 5-HT acted appreciably only through MOD-1 to increase steady-state dwelling at the expense of exploration on bacterial food [20].

**Fig. 7:**
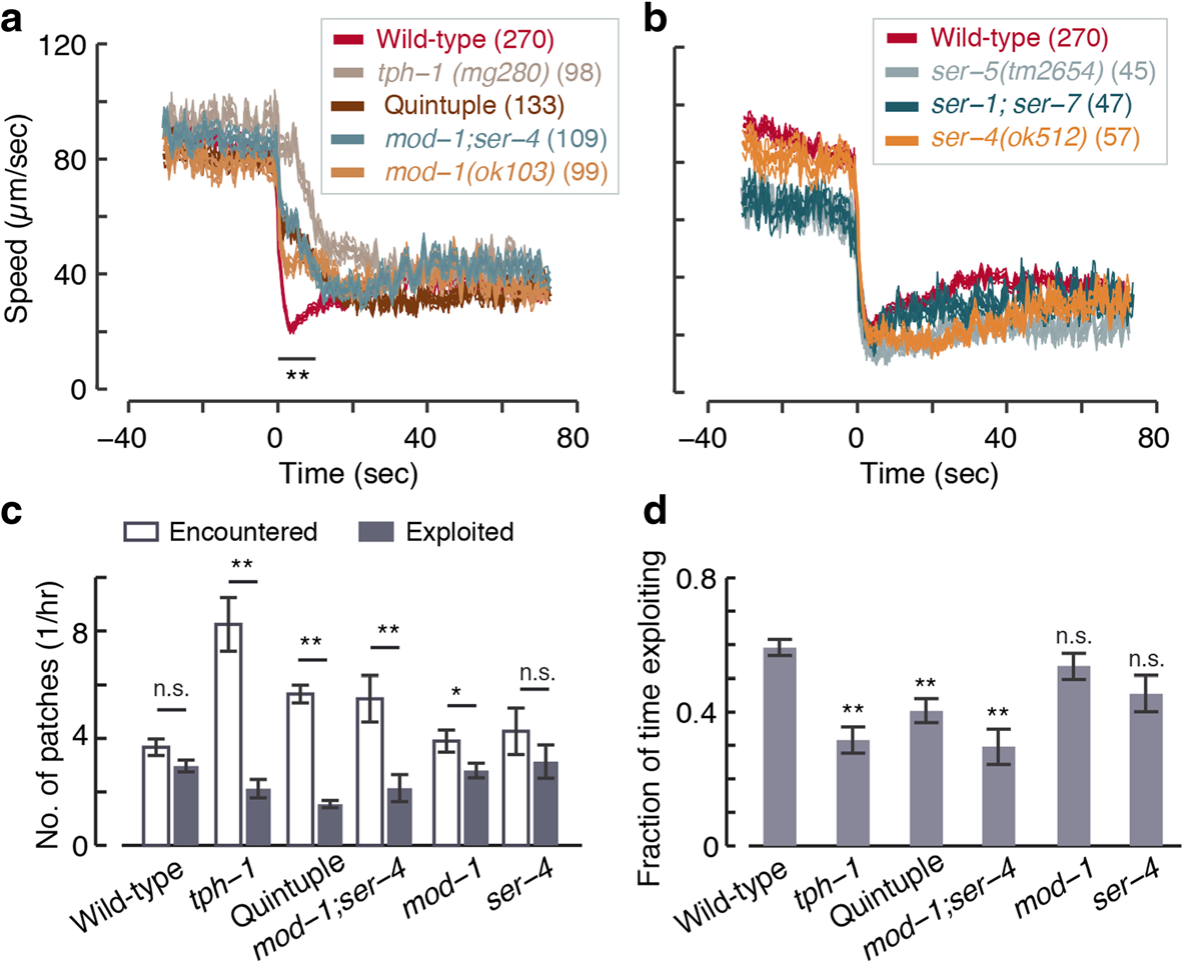
The MOD-1 5-HT-gated chloride channel is the primary mediator of abrupt slowdown upon encountering food. (**a**) The mean speeds of 5-HT receptor mutants deficient in 5-HT signaling encountering the edge of a bacterial lawn. Wild-type and *tph-1* same day controls are shown for comparison. (**b**) Same as panel (**a**) for 5-HT receptor mutants that did not negatively affect the abrupt slowdown upon encounter. (**c**) The number of patches encountered and exploited during 2.5 hours of foraging on a 5 × 5 hexagonal lattice of food patches. Mutants lacking all five serotonin receptors (quintuple) and *mod-1; ser-4* double mutants exhibited a strong defect that resembled *tph-1* mutants. Single receptor mutants exhibited a mild (*mod-1*) or no (*ser-4*) defect. (**d**) The overall fraction of time spent exploiting food resources in the experiments described in panel (**c**). Likewise, this criterion suggests that *tph-1*, the quintuple mutant, and *mod-1; ser-4* are mutants similarly deficient, while *mod-1* and *ser-4* mutants are similar to wild-type. Thick and thin lines or bars and error bars depict mean ± SEM, respectively. In panels (**a**, **b**), the number of animals assayed is noted in parentheses for each strain, mean velocities at t = 3–5 sec were compared to wild-type using an ANOVA test corrected post hoc for multiple comparisons using Tukey’s HSD test, and double asterisks denote a significant difference (*P* <0.01). Pairwise comparisons in panel (**c**) were performed using t-tests and the means in panel (**d**) were compared to wild-type using an ANOVA test corrected post hoc for multiple comparisons using Tukey’s HSD test. Single and double asterisks denote significant differences (*P* <0.05 and *P* <0.01, respectively). N (wild-type) = 19, N (*tph-1*) = 9, N (quintuple) = 7, N (*mod-1; ser-4*) = 8, N (*mod-1*) = 8, and N (*ser-4*) = 8

To test which receptors may mediate rapid decision-making upon newly encountering food we assayed 5-HT receptor mutants. In all cases, encounters were compared to same day wild-type and *tph-1* controls. On standard large lawns, quintuple mutants carrying null mutations of all five 5-HT receptors [26] were deficient in abrupt slowdown despite a slight but detectable initial response (Fig. 7a). As discussed above, the residual responsiveness of the quintuple mutants was insufficient for efficient exploitation of a complex environment (see Fig. 3). The encounter dynamics of *mod-1; ser-4* double mutants were identical to those of the quintuple mutants (Fig. 7a), while *ser-5* mutants and *ser-1; ser-7* double mutants exhibited wild-type-like abrupt slowdowns (Fig. 7b). In this assay, *mod-1* mutants exhibited a defect that was similar but not identical to that of *mod-1; ser-4*, and *ser-4* mutants resembled wild-type (Fig. 7 and Additional file 13: Figure S7).

To test the efficiency of foraging of these mutants in a complex environment we assayed them for 1.5–2.5 hours on a 5 × 5 patch arena. In this assay, the quintuple receptor mutants and *ser-4; mod-1* double mutants exhibited severe defects in the number of exploited patches (as compared to the number of patches encountered) and in the total time spent on exploitation during the assay. The phenotypes of these two strains were identical and resembled that of *tph-1* mutants (Fig. 7c, d). In contrast, *mod-1* and *ser-4* single mutants exhibited a milder defect and no significant defect, respectively. Combined, these results implicate MOD-1 and SER-4 in acting synergistically to mediate rapid decision-making.

## Discussion

Foraging and exploitation in patchy environments is a well-studied neuroethological paradigm for the enhancement of fitness afforded by decision-making circuits. Similarities in the challenges facing different species yield repeated algorithmic aspects of their solutions and conserved underlying mechanisms. As a result, decision-making is associated with fundamental functions of nervous systems [31, 43, 56]. Recently, the activity of dorsal raphe neurons was recorded in behaving mice that predicted and responded to rewards and punishment. The results indicated that serotonergic neurons signal about reward and punishment on short (hundreds of milliseconds) and long (minutes) timescales [57].

We have shown that in *C. elegans* 5-HT signaling accelerates the attenuation of locomotion upon encountering newly discovered food. We address the importance of this acceleration by showing that deficiencies in serotonergic signaling reduced the efficiency of foraging in a patchy environment. Previously, NSM was the major neuron implicated in affecting locomotion on food. It was shown to release serotonin extrasynaptically to diffuse in the vicinity of the nerve ring and activate a distributed circuit of target cells (as opposed to post-synaptic partners) [20]. Here, the serotonergic neuron types NSM and ADF were found to play complementary roles, where one neuron could partially compensate for a deficiency in another. The combined action of the 5-HT-activated chloride channel MOD-1 and the 5-HT receptor SER-4 was required for the accelerated response to discovering food.

Serotonergic modulation of locomotion has been examined under various sets of conditions [16, 18, 20, 22, 23, 28–30, 42, 58–61]. In contrast, fitness consequences of these effects on locomotion have not been studied in detail [31, 38]. Exogenous 5-HT robustly attenuates locomotion [22, 23] and stimulation of endogenous 5-HT release can cause slowdown [20, 28]. In addition, serotonergic signaling accelerates food-dependent aversive responses [30, 60, 61].

Prior to the isolation of *tph-1* mutants, Sawin et al. assayed locomotion by manually scoring body bends 5 min after placing animals on a bacterial lawn. Under these conditions, they showed that the slowdown of locomotion on food is enhanced in starved nematodes as compared to well-fed controls [16]. Enhanced slowdown was more severe on a *mod-5* mutant background and required the action of the MOD-1 receptor [28, 42, 58, 59]. The same assay was used to show that SER-4 contributed to post-starvation slowing on food [28] and to assay *tph-1* mutants [28, 62]. Both studies found that the slowdown of *tph-1* mutants was only mildly reduced as compared to wild-type and Gürel et al. did not report on the basal slowing of well-fed animals in their hands. These assays resulted in highly active, well-fed animals on food (compare basal slowing in [16, 19, 62] to steady-state locomotion on food in [20, 21]) suggesting that the transfer to the assay lawn may have been a factor in determining behavioral outcomes (as noted in [62]).

Unless specified otherwise, animals in the current study were deprived of food prior to the assay. However, in our hands starvation did not appreciably alter the preemptive slowdown, the abrupt slowdown upon encounter, nor the velocity 15 min post-encounter (Additional file 2: Figure S1). Moreover, 5-HT signaling deficiencies affected the approach to the edge of a lawn and the abruptness of responding to the encounter, but not center of mass motion 10–20 min thereafter. Additional file 2: Figure S1 and Additional file 3: Figure S2 demonstrate that the 5-min interval used in [28, 42, 58, 59, 62] is intermediate between the abrupt response to the encounter and the relaxation time to steady-state locomotion on food. Since the transient adaptation of locomotion upon encounter is not completed within 5 min, we suggest a different interpretation of the existing collective body of data: upon encountering food, rather than determining the target locomotion activity on food, serotonergic signaling accelerates the response to the sudden change in the external environment. The resulting abrupt response contributes to the efficiency of foraging in complex landscapes.

Flavell et al. assayed well-fed animals for 90 min on food, where wild-type animals predominantly dwell (as opposed to predominantly roaming off food). They found that the serotonergic NSM neurons are active during dwelling periods and inhibit roaming through 5-HT secretion. Moreover, they found that *tph-1* mutants roamed 3–5 fold more than wild-type on a bacterial lawn. Thus, serotonergic signaling can affect steady-state transitions between dwelling and roaming [20]. We did not observe large differences between the center of mass velocities of wild-type animals and *tph-1* mutants 5–20 min post-encounter (Additional file 3: Figure S2). However, since the focus of this work was on the encounter with food, the duration of our assays was limited as compared to the 90-min recordings with no prior starvation described in [20].

The NSM neuron is physically separated from the locomotor circuit by the basal membrane [63]. Based on its location and structure, NSM was hypothesized to play a role in reporting the availability of resources [64]. Nevertheless, it was implicated in modulating locomotion in [20, 28] and in the current study. Moreover, our data reveals prominent activation of NSM upon encountering bacteria. This raises the question of whether food signals onto NSM to drive locomotion through sensory neurons or whether NSM may directly monitor the state of the pharynx. Indirect activation of NSM was demonstrated in [45]. However, a detailed anatomical dissection of NSM identified a putative sensory process that may monitor pharyngeal activity [64]. We observed NSM activation in *osm-6* mutants, where cilium structure (and hence chemosensory function) is severely disrupted (Fig. 4c). Although our data cannot support a definitive answer, it is consistent with the notion that NSM monitors the state of the pharynx.

Significant physiological activity in ADF was detectable prior to the encounter with the food, while NSM responded detectibly upon encounter. Thus, ADF may act to refine the dynamics of the slowdown response. This can conceivably occur in several, not mutually exclusive, manners: 1) early release of 5-HT may prime the downstream neural circuit such that an abrupt slowdown can take place before NSM is fully activated; 2) the mild ADF-dependent early slowdown may positively affect detection (e.g. by affecting the angle of attack or providing more time for sampling the environment in the vicinity of food); and 3) responses to potentially competing stimuli may be suppressed in the vicinity of a patch of food. In addition, 5-HT released by ADF may contribute to the robustness of the response if it can be scavenged and used by *mod-5*-expressing neurons (NSM and/or others [29]).

Rather than identifying distinctive roles of individual serotonergic neuronal types, our combined physiological and behavioral data suggest partial functional redundancy. The phenotypes of wild-type animals, *tph-1* mutants, and *Ptph-1::TeTx* transgenics were consistent across conditions. Disrupting the function of ADF or NSM resulted in partial defects as compared to *tph-1* mutants. These findings suggest that different neurons capable of synthesis and/or uptake of 5-HT may act redundantly to modulate locomotion upon encountering food. Jafari et al., using the assays described in [16], reported that synaptic release of 5-HT from uptake neurons (that do not synthesize 5-HT themselves) was not required for locomotor responses to food deprivation. Rather, these neurons prevent exaggerated responses by scavenging extrasynaptic 5-HT [29]. A detailed analysis of neurons that promote exploitation in complex environments would require the dissection of 5-HT uptake, storage, and synaptic release in subsets of serotonergic and 5-HT uptake cells.

The SER-4 receptor and the MOD-1 chloride channel were shown to affect locomotion through GOA-1 activation in target cells or hyperpolarizing them, respectively [20, 28, 65, 66]. Head sensory- and inter-neurons were implicated as sites of action of GOA-1 and MOD-1 for inhibiting locomotion [20, 28]. It was further proposed that additional identified effectors act to facilitate the MOD-1 or SER-4 signaling pathways [28] or to oppose them [20]. While our data implicate SER-4 and MOD-1 in the abrupt response, the phenotypes of the *mod-1; ser-4* double and the quintuple receptor mutants fall short of recapitulating in full the deficits of *tph-1* mutants and *Ptph-1::TeTx* transgenics. Thus, our data suggests that an effect of a serotonin noncanonical receptor, metabolite, or precursor is yet to be accounted for.

When *C. elegans* navigate on shallow chemical or thermal gradients, typical responses to external cues include gradual changes in the curvature of their trajectories [67, 68] or modulation of the rate of infrequent sharp turns [17, 69, 70]. As a result, sensory stimuli can be integrated over timescales of tens of seconds, allowing the animal to filter out perceived abrupt (noisy) stimuli. In contrast, in a patchy environment an abrupt change in the availability of food is information of the utmost importance [71]. Correspondingly, behavioral responses must be rapid—analogous to a filter with a high cut-off frequency. The typical scales of the problem are expected to determine the required abruptness.

Typical roaming velocities off food are in the 100–200 μm/sec range. In our hands, patches as small as 400–500 μm in diameter merited exploitation. An order of magnitude estimate yields a timescale of slowdown of 2–3 sec, in agreement with our experimental observations and with the 2 Hz frequency of exploratory head motion [72]. Additional factors, such as concentration and quality, can affect the detailed responses to discovering food. However, abruptness remains advantageous under a range of conditions. Taken together, the robust activation of serotonergic neurons, the contribution of 5-HT re-uptake to proper locomotion dynamics, and the importance of this pathway to efficient foraging suggest that responding to newly discovered food is a key role of serotonergic signaling in *C. elegans*.

## Conclusions

This study suggests that a key role of serotonergic signaling in *C. elegans* is to accelerate decision-making and promote efficient exploitation of resources in complex environments. It primarily implicates the serotonergic neuron type NSM, the serotonin-gated chloride channel MOD-1, and the ortholog of mammalian 5-HT1 metabotropic serotonin receptors SER-4 in mediating this process. In addition, it demonstrates how different cells can use a common modulator to affect locomotion in complementary manners.

## Methods

### Strains

*C. elegans* strains were maintained and grown according to standard protocols [73]. The strains used in this study are listed in Additional file 14: Table S1.

### Behavioral assays: large bacterial lawn

To assay re-feeding on a large bacterial lawn, 72–96-hour-old adults were washed twice in a drop of M9 buffer and transferred to unseeded NGM plates for 1–2 hours. Animals were assayed on 35 mm NGM plates seeded with a 25 μl drop of 5x concentrated overnight culture of OP50 bacteria. The bacteria were spread in an oval shape of an area 100–150 mm^2^ on one side of the plate and assay plates were incubated overnight at 37 °C and kept at 4 °C prior to the assay. In each assay, 8–10 animals were transferred to M9 droplets on the empty side of the assay plate and started to crawl once the droplets were absorbed. Unless stated otherwise, animals that reached the bacterial lawn in less than 150 sec (<20 % of the total number of animals) were not scored in order to minimize the effect of the transfer on the data. Images were captured using a cooled CCD camera (CoolSNAP HQ2, Photometrics, Tucson, AZ, USA), an Olympus SZX16 stereomicroscope equipped with an SDF PLAPO 1XPF Objective (Olympus America Inc., Center Valley, PA, USA), and Micro-Manager Open Source Microscopy Software. Imaging was performed at 5 frames per sec, using a magnification of 0.7x and 4 × 4 binning. Under these conditions, the area of a single animal was 40–45 pixels. The centers of mass of the animal bodies were tracked using custom MATLAB scripts (MathWorks Inc., Natick, MA, USA). Center of mass velocities were computed using a temporal resolution of 1 sec. Each genotype was assayed at least on three different days and control animals were recorded each day. In our hands, day-to-day variability was typically small. To assess whether the preemptive slowdown was statistically significant, a linear function was fitted to the speeds of individual animals during the 50 sec prior to encountering food using the MATLAB Curve Fitting Toolbox. Post-encounter decay constants (τ) were based on a single exponential fit of the 0–150-sec data. The goodness of fit (R^2^) was higher than 0.9 in all cases. Experiments were performed on at least three independent days and day-to-day inconsistencies were not identified.

### Behavioral assays: micro-patches

For Additional file 5: Figure S4A, the 1x concentration was defined as OD_600_ = 1.5. Patches were arranged in concentric rings, 20 animals were placed on the assay plate, and only first encounters with a single patch were scored. Four ordinal categories were scored by visual inspection: 1) a full stop at the edge of the patch; 2) a noticeable slowdown of locomotion at the edge without a full stop; 3) a delayed slowdown of locomotion; and 4) the absence of a noticeable response. The former two response types resulted in feeding on the patch of food and the latter two did not.

For long-term assays in a complex environment, patches contained OP50 bacteria at a concentration of OD_600_ = 2.5. Assay plates were freshly seeded prior to the experiment. Individual food patches were seeded by gently touching preloaded thin gel loading tips to a standard agar plate, typically resulting in a patch diameter of 0.8–1 mm. Unless stated otherwise, the bacteria mix was supplemented with 10x kanamycin to prevent growth. Prolonged experiments (5–10 hours, Fig. 3) were performed on a 7 × 7 square lattice. Shorter experiments (2.5 hours, Fig. 7) were performed on a 5 × 5 hexagonal lattice. In both cases the distance between nearest neighbors was 2.5 mm. Food deprivation was performed as described above.

A single animal per plate was imaged at 5 frames per sec using a 5MP scientific CCD camera (Prosilica GC2450, Allied Vision Technologies, Stadtroda, Germany) at magnifications of 0.33–0.65x. A 3D printed square frame (18 mm inner and 24 mm outer diameter) lined with copper tape was used to contain the animal in the field of view. Custom LabVIEW (National Instruments Inc., Austin, TX, USA) and MATLAB scripts were used for image acquisition and data analysis.

Images were analyzed using the posture-based method as described previously [74]. Patch outlines were traced manually for each experiment, and frames where the nose-tip of the worm was within 5 % of the body length from a patch were labeled “encounter”. If, during an encounter, the mean velocity of the animal was at the lowest 15th percentile of measured roaming velocities then the frames of the encounter were labeled “exploitation”. Brief (exploratory or accidental) excursions out of the patch, in which the distance between the edge and the nose-tip was no greater than 20 % of the body length, were consolidated. Thus, motion on the order of 100 μm beyond the patch edge did not fractionate a continuous event. Patch coverage was quantified by determining the area occupied during each exploitation event. Experiments were performed on at least three independent days and day-to-day inconsistencies were not identified.

### Calcium imaging

Calcium imaging was performed on freely behaving animals on large bacterial lawn, 60 mm diameter, assay plates. Here, 50 μl overnight bacterial culture was seeded in a ring and incubated overnight at 37 °C. The transgenic strains assayed expressed the calcium indicator GCaMP3.0 in the target neuron on a *lite-1* mutant background [53, 54] (see Additional file 14: Table S1). Food deprivation was performed as described above, several animals were initially placed at the center of the ring, and a single animal approaching the inner diameter of the ring was continuously imaged. To image NSM during evoked pauses (Fig. 4, left inset), a standard platinum pick was manually placed near the anterior of a forward moving animal such that it gently touched it due to its own motion.

The analysis of neuronal calcium transients was performed similarly to the previously described procedure in [75]. Data shown in Fig. 4a and Additional file 11: Figure S5 was acquired using the setup described above for large bacterial lawn assays. Imaging was performed at 5 frames per sec at a magnification of 10x for up to 25 min. In our hands, bacterial lawns had clearly visible sharp edges. First-day adults were transferred to assay plate and allowed several minutes to equilibrate. Animals were tracked manually until they were approximately 15 body lengths from the edge of the food patch, at which point the assay plate remained in place. For reverse patch imaging (Additional file 11: Figure S5A), assay plates were prepared with 2 ml agar; the resulting thickness was 2–3 mm. Prior to the assay, the agar was carefully flipped such that the animals crawled on the agar surface opposite to the lawn. The minimal possible distance of the animal from the bacteria was thus 2–3 mm.

For data shown in Fig. 4b, c, imaging was acquired at a frame rate of 11 frames per sec. Images were captured using a cooled EMCCD camera (Evolve 512, Photometrics), an Olympus 83x inverted microscope equipped with a UPLSAPO 10x objective (Olympus America Inc.), and Micro-Manager Open Source Microscopy Software. Animals were tracked automatically using a custom worm tracker (coded in MATLAB).

Image analysis was performed using custom MATLAB scripts, which identified the fluorescent neuron (the only bright particle in the head), measured the background fluorescence in its vicinity, and calculated the background subtracted mean fluorescence intensity of the identified particle. Velocities in Fig. 4b were calculated based on the displacement of the imaged cell. Experiments were performed on three independent days and day-to-day inconsistencies were not identified.

### Optogenetics

Optogenetic assays were performed in 1.1 × 1.1 mm microfluidic chambers in which the animals could move freely. Blue light illumination (λ = 475 ± 15 nm) was supplied by a Luxeon Star 7-LED assembly (Luxeon Star LEDs, Brantford, ON, Canada) with a diffused optic array driven by a 700 mA FlexBlock driver (LEDdynamics, Inc., VT, USA). The LED assembly was mounted to the scopes approximately 7 cm from the sample location. Light intensity at the location of the animals was set to 0.8 mW/mm^2^. Transgenic animals expressing the light-gated ion channel channelrhodopsin (ChR2) in all serotonergic neurons on a *lite-1* mutant background (see Additional file 14: Table S1) were illuminated for 30 sec in the presence of 1 mM ATR. Control animals were assayed in the absence of ChR2 and presence of ATR (Fig. 6). The level of activity of the animals was assayed using the frame subtraction method as previously described [55]. In order to obtain the timescales of slowdown and recovery, as described in the text, a single exponential function was fitted to each dataset using the MATLAB curve fitting toolbox. The goodness of fit (R^2^) was higher than 0.9 in all cases except for the recovery of the faster recovering *lite-1;* Ex[*Ptph-1::ChR2*] line (shown in orange), where R^2^ = 0.73. Experiments were performed on three independent days and day-to-day inconsistencies were not identified.

### Statistical analysis

Data analysis was performed using custom MATLAB scripts. Individual statistical tests are detailed in the corresponding figure legends.

## Additional files

Additional file 1:
**Movie S1.** Serotonin accelerates the response to the edge of a bacterial lawn. An example of wild-type animals (left) and *tph-1(mg280)* mutants (right) encountering a large lawn of bacterial food. The wild-type response to the encounter was visibly more abrupt. The movie was sped up 50x as compared to real-time. (AVI 24296 kb) Additional file 2: Figure S1. (A, B) The center of mass velocities of wild-type animals that were kept off food for 2 hours or 5 min prior to the assay. In our hands, the different starvation conditions did not strongly affect the abrupt slowdown upon encountering food or locomotion thereafter. A small but significant difference was observed in baseline velocities (as determined using a t-test, *P* <0.01). (C–F) Scatter plots of wild-type behavior: velocities at t = 3–5 sec post-encounter; velocities 1,000–1,300 sec post-encounter; the preemptive slowdown during the 50 sec prior to the encounter; and the relative velocities post-encounter. Filled/dark circles represent all animals that arrived at the edge of the bacterial lawn at least 150 sec after being transferred to the assay plate. All four aspects of locomotion are weakly or not significantly correlated with the duration of prior food deprivation (denoted by R). Empty/light circles denote animals that arrived at the edge of the lawn less than 150 sec after being transferred to the assay plate. When these data are added to the analysis, correlations (denoted by r) with the duration of prior food deprivation remain weak or insignificant. (TIF 578 kb) Additional file 3: Figure S2. (A, B) The same assays and analysis as described in Fig. 2a, b were also performed on a *mod-5* mutant background. (C) The velocity of wild-type animals, *tph-1* mutants, *mod-5* mutants, and transgenics in which the serotonergic neurons have been genetically silenced as measured up to 1,500 sec after the encounter with food. (D) The same assays and analysis as described in Fig. 2a, b are shown for the three independent transgenic lines. (E, F) The same assays and analysis as described in Fig. 2a, b were also performed on *bas-1* mutants, lacking serotonin and dopamine, and *cat-2* mutants, lacking dopamine. Dopamine in and of itself was not required for rapid decision-making upon encountering food. Comparisons were performed using an ANOVA test corrected post hoc for multiple comparisons using Tukey’s HSD test. Single and double asterisks denote significant differences (*P* <0.05 and *P* <0.01, respectively). (TIF 541 kb) Additional file 4: Figure S3. The relative velocities of the key mutants and transgenics assayed in this work. Relative velocities were calculated as the ratio of the mean velocities at t = 3–5 sec and t = −60–(-50) sec, where the encounter was defined as t = 0. Comparisons were performed using an ANOVA test corrected post hoc for multiple comparisons using Tukey’s HSD test. Single and double asterisks denote significant differences (*P* <0.05 and *P* <0.01, respectively). (TIF 396 kb) Additional file 5: Figure S4. (A) Responses of wild-type and 5-HT-deficient strains upon encounter with a micro-patch (see Methods). The number of animals assayed for each strain is noted in parentheses. Comparisons were performed by assigning the four types of responses numerical values (0–3) and using an ANOVA test corrected post hoc for multiple comparisons using Tukey’s HSD test. Single and double asterisks denote a significant difference from wild-type (*P* <0.05 and *P* <0.01, respectively). Here, a single *Ptph-1::TeTx* line was assayed. (B) Sample maps of two patchy environments on which a single wild-type animal (left) and a single *tph-1* mutant (right) were assayed. Each outlined circle depicts a single patch of bacterial food. Cyan, blue, red, and yellow colors depict the positions of the nose of the animal during single exploitation events. Grey depicts events in which the animal encountered the patch but did not slow down sufficiently to be considered “exploiting” for the purpose of the analysis. (C, D) The efficiency of exploitation in the small patch assay of TeTx transgenics and Cre-mediated *tph-1* deletion strains. Functional ablations of individual neurons resulted in partial phenotypes. Deletion of *tph-1* in NSM and ADF resulted in a mild partial defect. (TIF 758 kb) Additional file 6:
**Movie S2.** Serotonin promotes exploitation of a single patch. A wild-type animal (left), a *tph-1* mutant (middle), and a transgenic animal in which serotonergic neurons were genetically silenced (right) encounter a small patch of food (D = 700–800 μm). Only the wild-type animal was able to efficiently exploit this resource. (AVI 14921 kb) Additional file 7:
**Movie S3.** Wild-type foraging in a complex environment. The midline of a wild-type animal (black curve, circle denotes the head) as it forages on a grid of bacterial patches (grey). Patches are circled with a blue ring if they were encountered and a yellow ring while they are being exploited. Colored areas depict individual exploitation events (as in Additional file 5: Figure S4). The movie was sped up 100x as compared to real-time. (MOV 7325 kb) Additional file 8:
**Movie S4.** A *tph-1* mutant foraging in a complex environment. An example of the behavior of a *tph-1(mg280)* mutant depicted as described for Additional file 7: Movie S3. (MOV 8207 kb) Additional file 9:
**Movie S5.** Physiological activity in NSM: the pharyngeal serotonergic neuron type. NSM is activated robustly and acutely upon encountering the edge of a newly discovered patch of food. (AVI 4421 kb) Additional file 10:
**Movie S6.** Physiological activity in ADF: the chemosensory serotonergic neuron type. ADF is activated prior to encountering the edge of a newly discovered patch of food. (AVI 7492 kb) Additional file 11: Figure S5. (A) Mean values of NSM::GCaMP (grey) and ADF::GCaMP (black) fluorescence from animals assayed on a reversed patch (see Methods). The time in which the animal crawled directly above the edge of the bacterial lawn was defined as t = 0 . Mean fluorescence was measured during 20-sec periods, before and around the mock encounter. Baseline activity was measured in the same neurons 30–50 sec prior to the mock encounter. The change in fluorescence observed in ADF mirrored the change observed several seconds prior to the encounter. Bars depict mean ± SEM, the number of animals assayed for each strain is noted in parentheses, pairwise comparisons were performed using a t-test, and the asterisk denotes a statistically significant difference between the two periods (*P* <0.05). (B) The mean kinetics of the GCaMP fluorescence data from Fig. 4a and the mean velocities from Fig. 2a. The yellow shaded area denotes post-encounter times (see Discussion). (TIF 116 kb) Additional file 12: Figure S6 Histograms of preemptive slopes measured in individual animals during the 50 sec prior to encountering a large patch of food. Negative slopes are emphasized in red. Errors denote ± SEM and *P* values denote the probability that the measured distribution of slopes was obtained from a distribution with zero mean, as determined by a t-test. (TIF 507 kb) Additional file 13: Figure S7. The mean speeds of animals carrying two independent *mod-1* alleles, assayed for encountering the edge of a bacterial lawn. The two mutants exhibited identical locomotion dynamics around the time of the encounter. (TIF 138 kb) Additional file 14: Table S1. Strains. (DOCX 18 kb)
