# Supplementary figures and images for: Serotonin promotes exploitation in complex environments by accelerating decision-making
Source: BMC Biol. 2016 Feb 4;14:9. doi: 10.1186/s12915-016-0232-y (PMC4743430; doi:10.1186/s12915-016-0232-y)

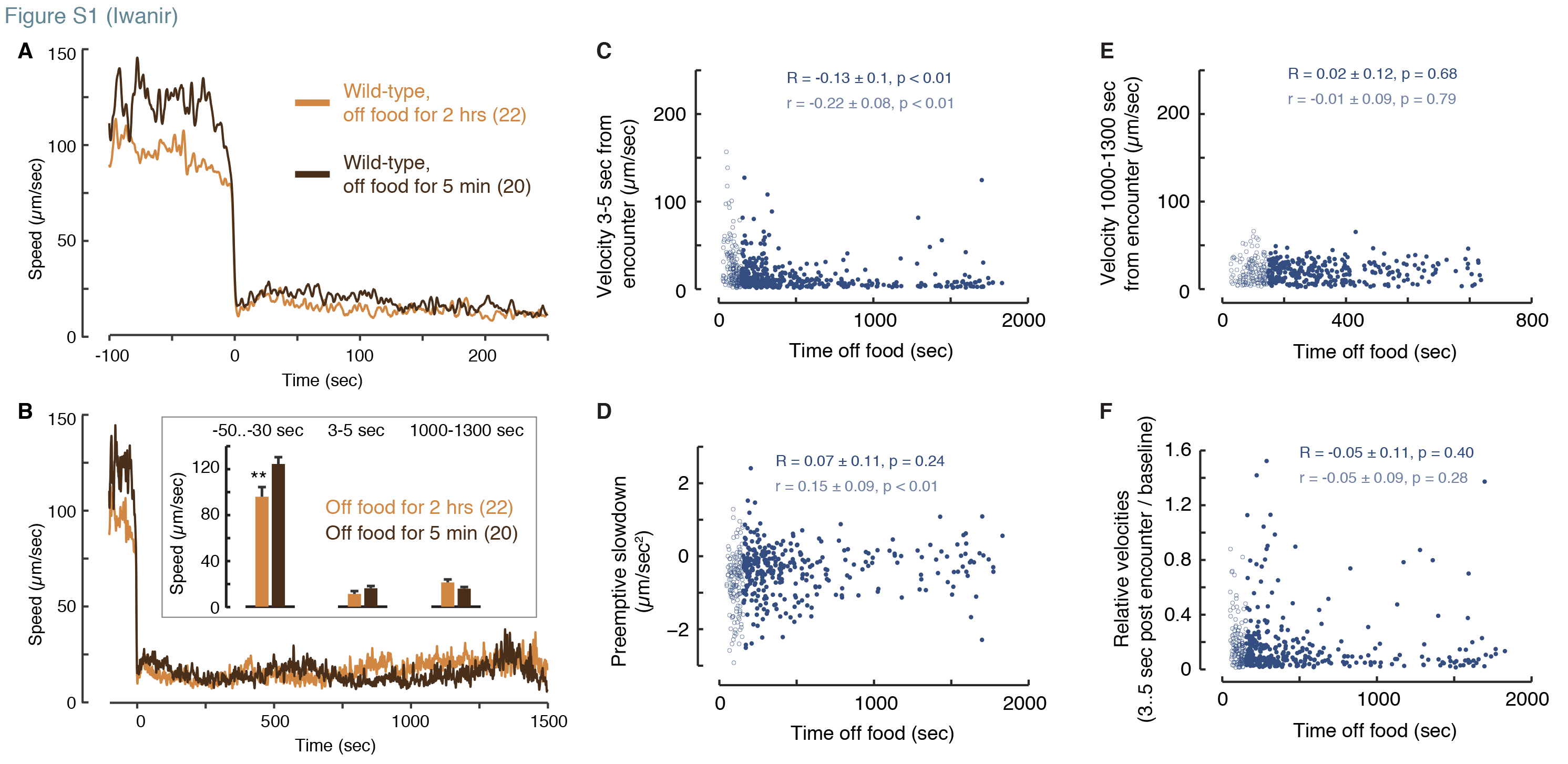

Supplement: Additional file 2: Figure S1. — (A, B) The center of mass velocities of wild-type animals that were kept off food for 2 hours or 5 min prior to the assay. In our hands, the different starvation conditions did not strongly affect the abrupt slowdown upon encountering food or locomotion thereafter. A small but significant difference was observed in baseline velocities (as determined using a t-test, P <0.01). (C–F) Scatter plots of wild-type behavior: velocities at t = 3–5 sec post-encounter; velocities 1,000–1,300 sec post-encounter; the preemptive slowdown during the 50 sec prior to the encounter; and the relative velocities post-encounter. Filled/dark circles represent all animals that arrived at the edge of the bacterial lawn at least 150 sec after being transferred to the assay plate. All four aspects of locomotion are weakly or not significantly correlated with the duration of prior food deprivation (denoted by R). Empty/light circles denote animals that arrived at the edge of the lawn less than 150 sec after being transferred to the assay plate. When these data are added to the analysis, correlations (denoted by r) with the duration of prior food deprivation remain weak or insignificant. (TIF 578 kb) [file 12915_2016_232_MOESM2_ESM.tif]

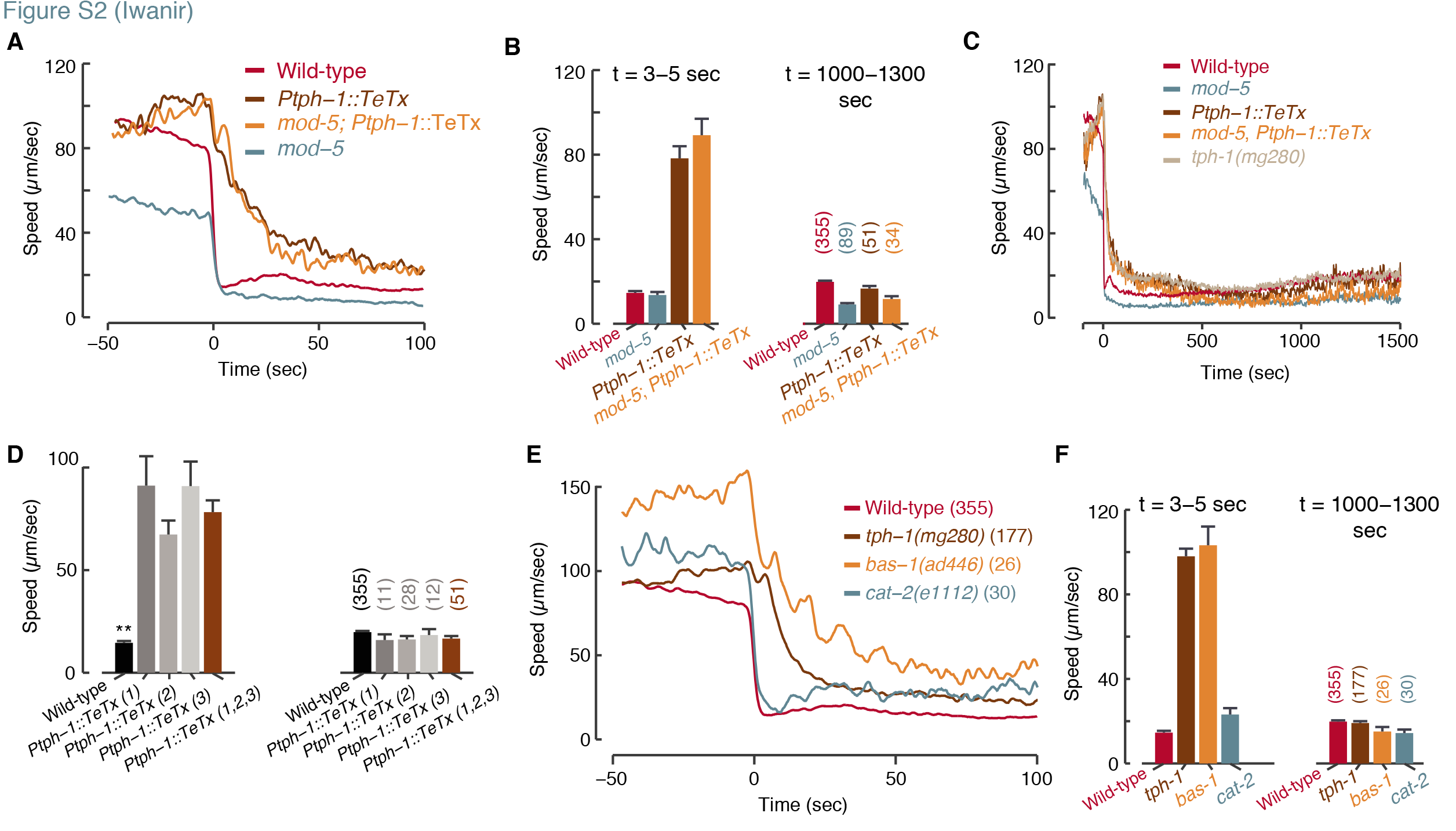

Supplement: Additional file 3: Figure S2. — (A, B) The same assays and analysis as described in Fig. 2a, b were also performed on a mod-5 mutant background. (C) The velocity of wild-type animals, tph-1 mutants, mod-5 mutants, and transgenics in which the serotonergic neurons have been genetically silenced as measured up to 1,500 sec after the encounter with food. (D) The same assays and analysis as described in Fig. 2a, b are shown for the three independent transgenic lines. (E, F) The same assays and analysis as described in Fig. 2a, b were also performed on bas-1 mutants, lacking serotonin and dopamine, and cat-2 mutants, lacking dopamine. Dopamine in and of itself was not required for rapid decision-making upon encountering food. Comparisons were performed using an ANOVA test corrected post hoc for multiple comparisons using Tukey’s HSD test. Single and double asterisks denote significant differences (P <0.05 and P <0.01, respectively). (TIF 541 kb) [file 12915_2016_232_MOESM3_ESM.tif]

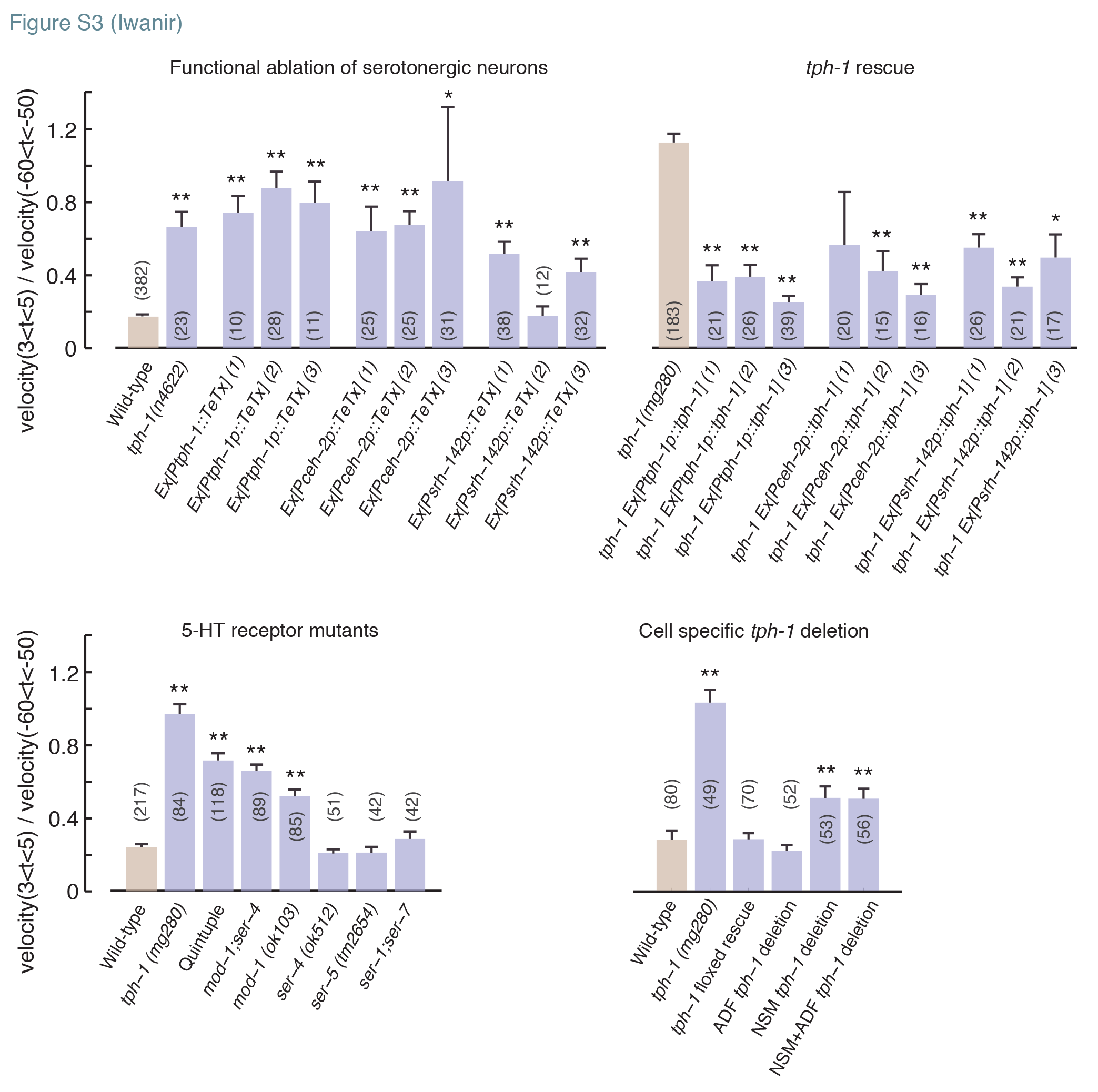

Supplement: Additional file 4: Figure S3. — The relative velocities of the key mutants and transgenics assayed in this work. Relative velocities were calculated as the ratio of the mean velocities at t = 3–5 sec and t = −60–(-50) sec, where the encounter was defined as t = 0. Comparisons were performed using an ANOVA test corrected post hoc for multiple comparisons using Tukey’s HSD test. Single and double asterisks denote significant differences (P <0.05 and P <0.01, respectively). (TIF 396 kb) [file 12915_2016_232_MOESM4_ESM.tif]

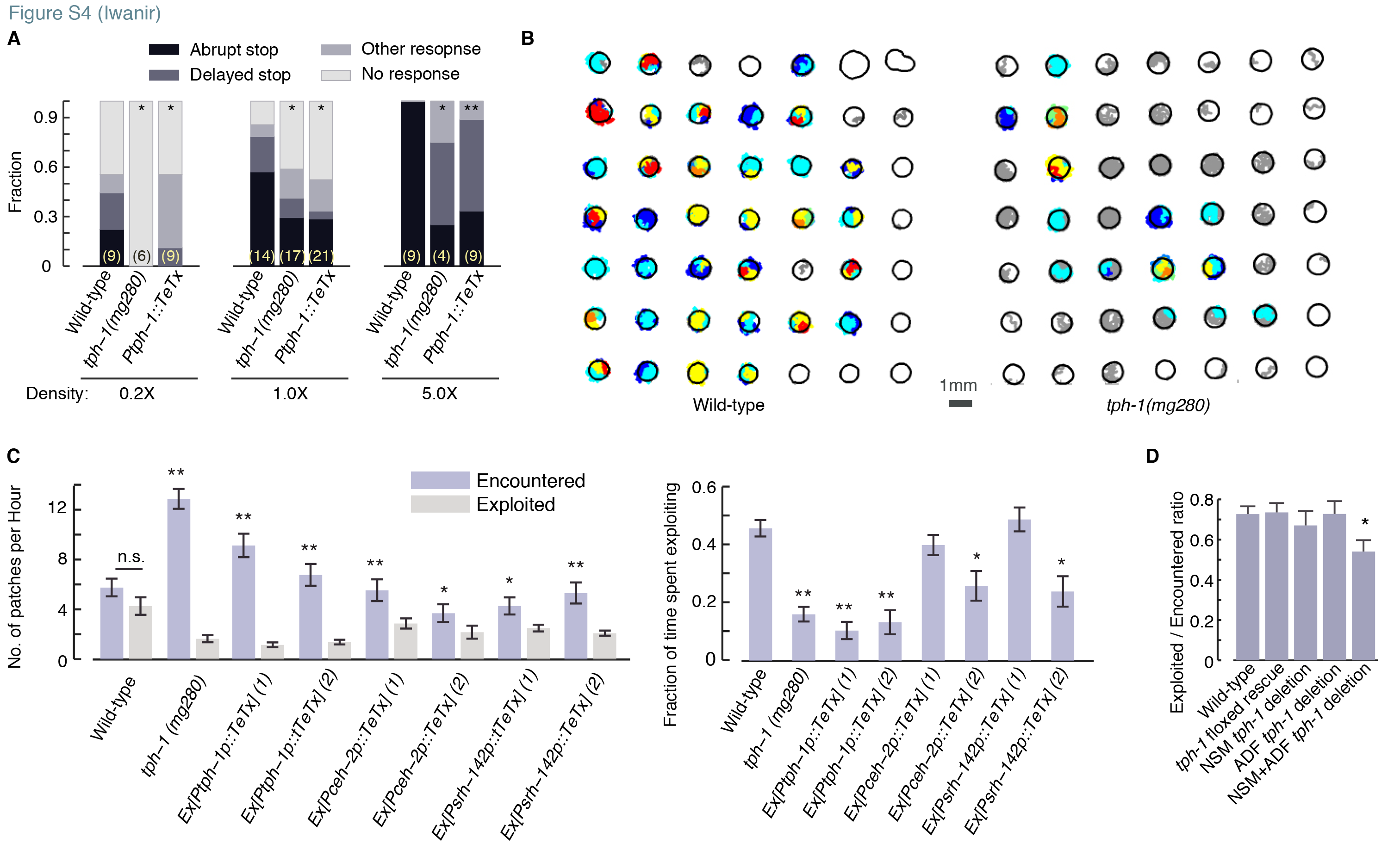

Supplement: Additional file 5: Figure S4. — (A) Responses of wild-type and 5-HT-deficient strains upon encounter with a micro-patch (see Methods). The number of animals assayed for each strain is noted in parentheses. Comparisons were performed by assigning the four types of responses numerical values (0–3) and using an ANOVA test corrected post hoc for multiple comparisons using Tukey’s HSD test. Single and double asterisks denote a significant difference from wild-type (P <0.05 and P <0.01, respectively). Here, a single Ptph-1::TeTx line was assayed. (B) Sample maps of two patchy environments on which a single wild-type animal (left) and a single tph-1 mutant (right) were assayed. Each outlined circle depicts a single patch of bacterial food. Cyan, blue, red, and yellow colors depict the positions of the nose of the animal during single exploitation events. Grey depicts events in which the animal encountered the patch but did not slow down sufficiently to be considered “exploiting” for the purpose of the analysis. (C, D) The efficiency of exploitation in the small patch assay of TeTx transgenics and Cre-mediated tph-1 deletion strains. Functional ablations of individual neurons resulted in partial phenotypes. Deletion of tph-1 in NSM and ADF resulted in a mild partial defect. (TIF 758 kb) [file 12915_2016_232_MOESM5_ESM.tif]

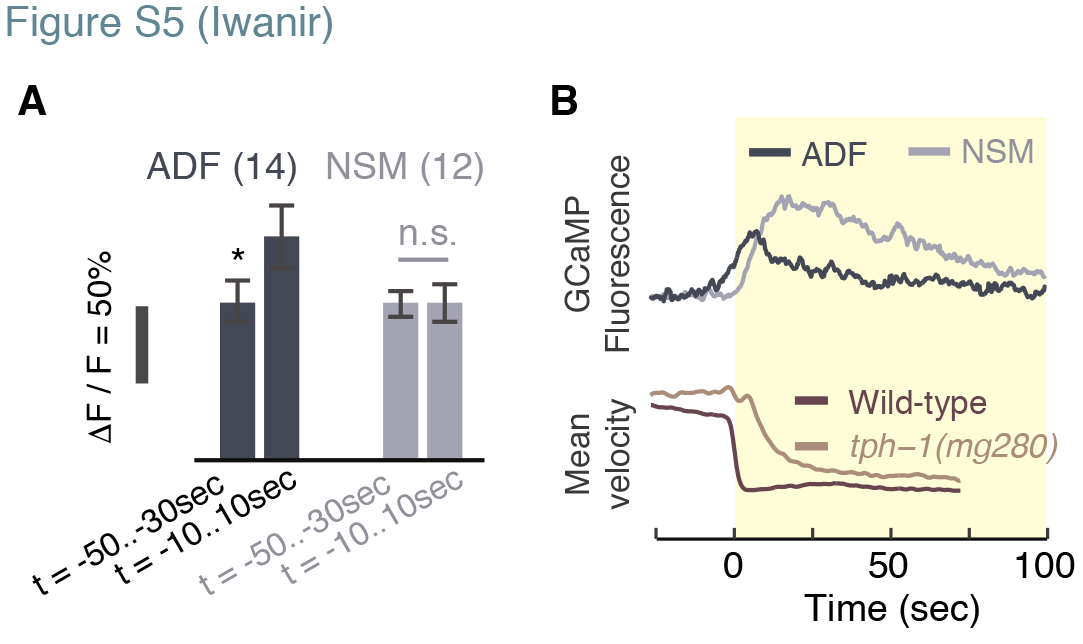

Supplement: Additional file 11: Figure S5. — (A) Mean values of NSM::GCaMP (grey) and ADF::GCaMP (black) fluorescence from animals assayed on a reversed patch (see Methods). The time in which the animal crawled directly above the edge of the bacterial lawn was defined as t = 0 . Mean fluorescence was measured during 20-sec periods, before and around the mock encounter. Baseline activity was measured in the same neurons 30–50 sec prior to the mock encounter. The change in fluorescence observed in ADF mirrored the change observed several seconds prior to the encounter. Bars depict mean ± SEM, the number of animals assayed for each strain is noted in parentheses, pairwise comparisons were performed using a t-test, and the asterisk denotes a statistically significant difference between the two periods (P <0.05). (B) The mean kinetics of the GCaMP fluorescence data from Fig. 4a and the mean velocities from Fig. 2a. The yellow shaded area denotes post-encounter times (see Discussion). (TIF 116 kb) [file 12915_2016_232_MOESM11_ESM.tif]

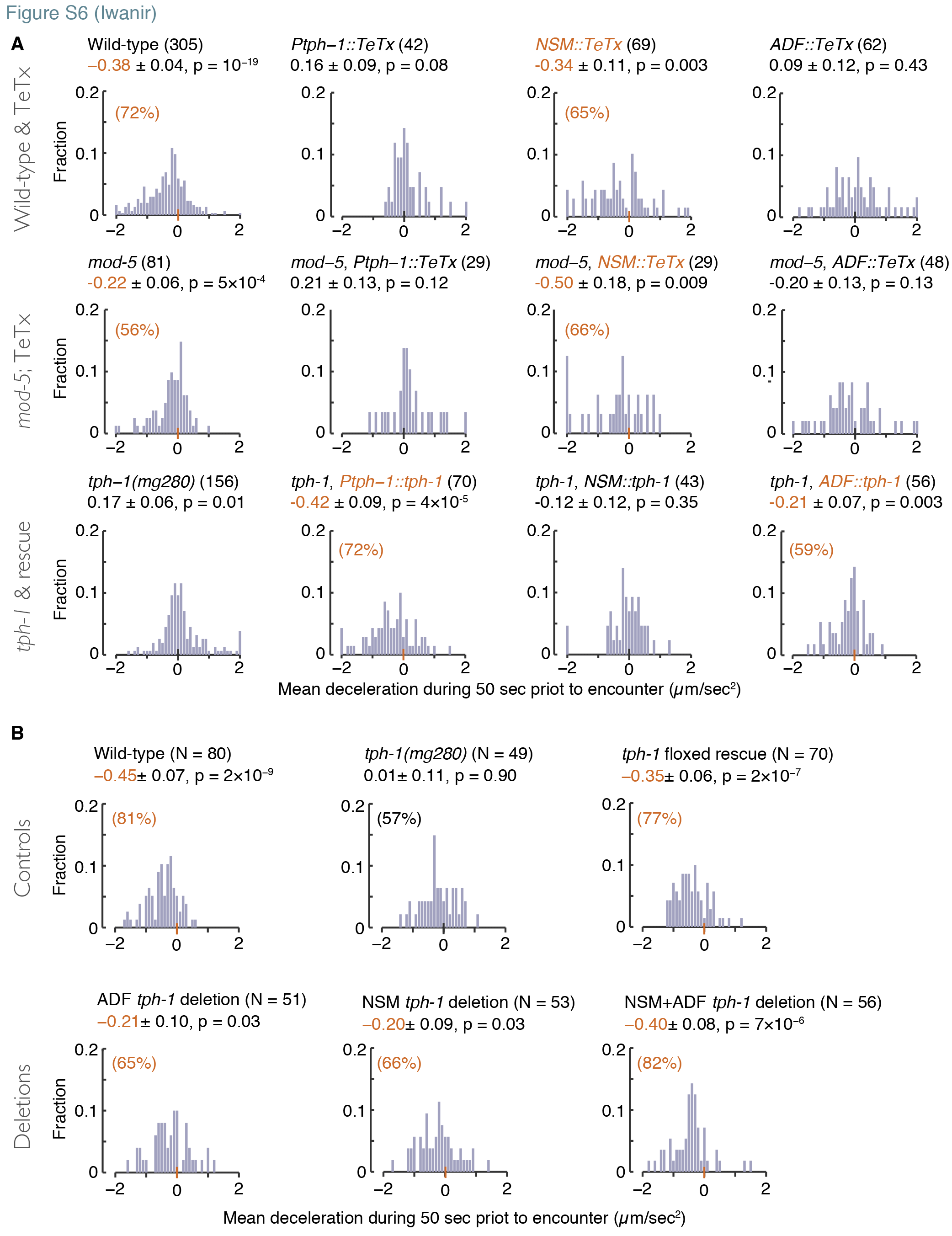

Supplement: Additional file 12: Figure S6 — Histograms of preemptive slopes measured in individual animals during the 50 sec prior to encountering a large patch of food. Negative slopes are emphasized in red. Errors denote ± SEM and P values denote the probability that the measured distribution of slopes was obtained from a distribution with zero mean, as determined by a t-test. (TIF 507 kb) [file 12915_2016_232_MOESM12_ESM.tif]

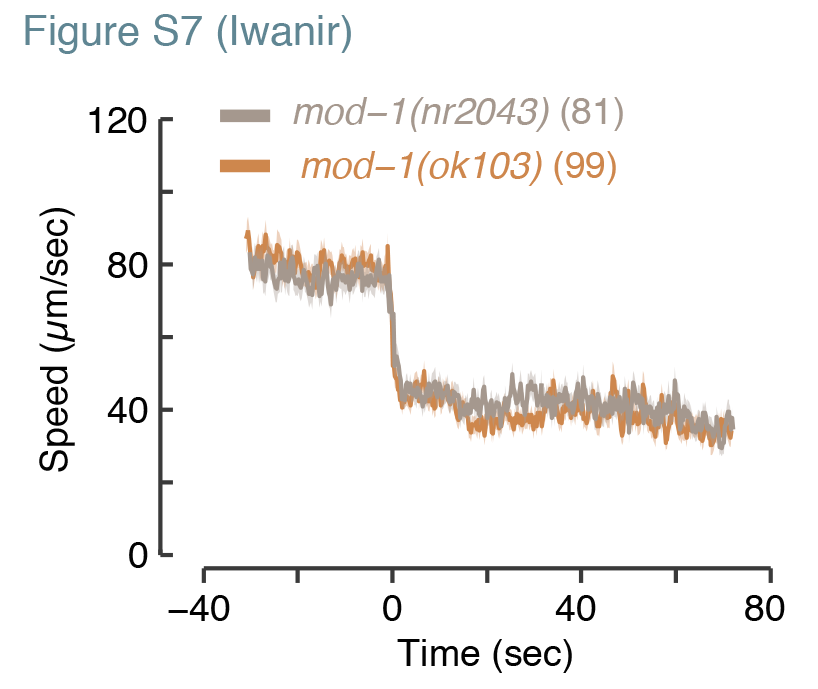

Supplement: Additional file 13: Figure S7. — The mean speeds of animals carrying two independent mod-1 alleles, assayed for encountering the edge of a bacterial lawn. The two mutants exhibited identical locomotion dynamics around the time of the encounter. (TIF 138 kb) [file 12915_2016_232_MOESM13_ESM.tif]
