# Supplementary material for: Serotonin promotes exploitation in complex environments by accelerating decision-making
Source: BMC Biol. 2016 Feb 4;14:9. doi: 10.1186/s12915-016-0232-y (PMC4743430; doi:10.1186/s12915-016-0232-y)
Supplement: Additional file 14: Table S1. — Strains. (DOCX 18 kb) [file 12915_2016_232_MOESM14_ESM.docx]

Table T1 – strains.

| **Name** | **Genotype** | **Source/Comments** |
| --- | --- | --- |
| N2 | wild-type | CGC |
| MT15434 | *tph-1(mg280)* | Horvitz lab; This line was backcrossed to remove satellite mutations affecting locomotion. |
| FQ77 | *tph-1(n4622)* | Ringstad lab |
| MT7988 | *bas-1(ad446)* | CGC |
| CB1112 | *cat-2(e1112)* | CGC |
| MT9668 | *mod-1 (ok103)* | CGC |
| MT9667 | *mod-1(nr2043)* | Koelle lab |
| AQ866 | *ser-4(ok512)* | CGC |
| RB2277 | *ser-5(ok3087)* | CGC |
| RWK213 | *ser-5(tm2654)* | Komuniecki lab |
| OT179 | *ser-4(ok512); mod-1(ok103)* | Komuniecki lab |
| DA2109 | *ser-7(tm1325); ser-1(ok345)* | Komuniecki lab |
| RWK3 | *mod-1; ser-1; ser-4; ser-5; ser-*7 quintuple mutant | Komuniecki lab |
| MT1082 | *egl-1(n487)* | CGC |
| MT2060 | *egl-1(n987)* | CGC |
| MT2251 | *Egl-1(1084)* | CGC |
| INV80003 | *mod-5 (n3314)* | 6x Backcrossed MT9772 |
| INV90001 | *mod-1 (ok103)*; *ser-4(ok512)* |  |
| INV33006  INV33009  INV33010 | N2; Ex[*ptph-1::TeTx-mCherry punc122::GFP]* | 1 kb promoter |
| INV30003  INV30004  INV30008 | N2; Ex[*pceh-2::TeTx-mCherry unc122::GFP]* | NSM specific; 1.6 kb promoter |
| INV30001  INV30002  INV30010 | N2; Ex[*psrh-142::TeTx-mCherry punc122::GFP]* | ADF-specific; 3.5 kb promoter |
| INV63006 | *mod-5(n3314);* Ex[*ptph-1::TeTx-mCherry punc122::GFP]* | 1 kb promoter |
| INV60008 | *mod-5(n3314);* Ex[*pceh-2::TeTx-mCherry punc122::GFP]* | NSM specific; 1.6 kb promoter |
| INV60001  INV60009  INV60010 | *mod-5(n3314);* Ex[*psrh-142::TeTx-mCherry punc122::GFP]* | ADF-specific; 3.5 kb promoter |
| INV70001  INV70002 | *tph-1(mg280);* Ex[*ptph-1::tph-1:: SL2::mCherry punc122::GFP]* | Native 1.6 kb promoter |
| INV70004 | *tph-1(mg280);* Ex[*ptph-1::tph-1:: SL2::GFP punc122::RFP]* | 1 kb promoter |
| INV70005  INV70006  INV70007 | *tph-1(mg280);* Ex[*pceh-2::tph-1:: SL2::GFP punc122::RFP]* | NSM specific; 1.6 kb promoter |
| INV70008  INV70009  INV70010 | *tph-1(mg280);* Ex[*psrh-142::tph-1::SL2::GFP punc122::RFP]* | ADF-specific; 3.5 kb promoter |
| INV50001 | *lite-1(ce314)*; Ex[*Ptph-1::mcherry::SL2::GCaMP3 punc122::RFP]* | 0.5 kb promoter; Expression detectable in NSM mainly |
| INV50002 | *lite-1(ce314*); Ex[*Psrh-142::mcherry::SL2::GCaMP3 punc122::RFP]* | ADF-specific; 3.5 kb promoter |
| AZ200 | *osm-6(p811); lite-1(ce314*); Ex[*ceh-2::mcherry::SL2::GCaMP3 punc122::RFP]* | 0.5 kb promoter; Expression detectable in NSM mainly |
| AZ201 | *osm-6(p811); lite-1(ce314*); Ex[*Psrh-142::mcherry::SL2::GCaMP3 punc122::RFP]* | ADF-specific; 3.5 kb promoter |
| INV60005  INV60006 | *lite-1 (ce314)*; Ex[*ptph-1:::Chr2-mCherry punc-122::GFP]* | 1 kb promoter |
| INV60012 | *mod-5(n3314); lite-1 (ce314)*; Ex[*ptph-1::Chr2-mCherry punc-122::GFP]* | 1 kb promoter |
| CX13228 | *tph‐1(mg280)*; kySi56[*floxed tph‐1 genomic rescue*] | Flavell et al., 2013. |
| CX13572 | *tph‐1(mg280);* kySi56 IV*;* kyEx4057[*ceh‐2::nCre*] | Flavell et al., 2013, NSM*tph-1*deletion |
| CX13571 | *tph‐1(mg280);* kySi56 IV*;* kyEx4077[*srh-142::nCre*] | Flavell et al., 2013, ADF*tph-1*deletion |
| CX13576 | *tph‐1(mg280);* kySi56 IV*;* kyEx4107[*egl‐6::nCre*] | Flavell et al., 2013, HSN*tph-1*deletion |
| CX15658 | *tph‐1(mg280);* kySi56 IV*;* kyEx5262[ceh-2::nCre, *egl‐6::nCre*] | Flavell et al., 2013, HSN and NSM*tph-1*deletion |
| ERL78 | *tph‐1(mg280);* kySi56 IV; opyEx18[*ceh‐2::nCre, srh‐142::nCre, myo-3::mCherry*] | Levine lab, Harvard University, ADF and NSM *tph-1* deletion |

* Lines were generated in the Biron Lab unless other source is mentioned.
